# Taste Genetics and Oral Health Symptoms in the Canadian Longitudinal Study on Aging

**DOI:** 10.3390/ijms27188128

**Published:** 2026-09-12

**Authors:** Marziyeh Shafizadeh, Vikram Bhatia, Samah Ahmed, Britt Drögemöller, Chrysi Papadimitropoulos, Philip St. John, Rajinder P. Bhullar, Prashen Chelikani, Carol A. Hitchon

**Affiliations:** 1 Department of Oral Biology and Manitoba Chemosensory Biology Research Group, Dr. Gerald Niznick College of Dentistry, Rady Faculty of Health Sciences, University of Manitoba, Winnipeg, MB R3E 0W2, Canada; mojan.shafizadeh@umanitoba.ca (M.S.);; 2 Children’s Hospital Research Institute of Manitoba (CHRIM), University of Manitoba, Winnipeg, MB R3E 0Z3, Canada; 3 Department of Biochemistry and Medical Genetics, Max Rady College of Medicine, Rady Faculty of Health Sciences, University of Manitoba, Winnipeg, MB R3E 3N4, Canada; 4 Paul Albrechtsen Research Institute CancerCare Manitoba, Winnipeg, MB R3E 0V9, Canada; 5 Centre on Aging, University of Manitoba, Winnipeg, MB R3T 2N2, Canada; 6 Department of Dental Diagnostic and Surgical Sciences, Dr. Gerald Niznick College of Dentistry, Rady Faculty of Health Sciences, University of Manitoba, Winnipeg, MB R3E 0W2, Canada; 7 Department of Internal Medicine, Max Rady College of Medicine, Rady Faculty of Health Sciences, University of Manitoba, Winnipeg, MB R3A 1R9, Canada

**Keywords:** taste receptor, taste genetics, pseudogene, oral health, CLSA, allelic association, single nucleotide polymorphism, temporomandibular disorder, arthritis

## Abstract

Bitter taste receptors (T2Rs) contribute to innate immune responses and taste preferences. Variants in T2R genes (*TAS2Rs*) may increase the risk of adverse oral health. This study investigated the association of single nucleotide polymorphisms (SNPs) in 25 *TAS2R* genes and 12 *TAS2R* pseudogenes with oral health symptoms within the Canadian Longitudinal Study on Aging (CLSA) cohort. Allele frequencies were calculated using PLINK and compared with the 1000 Genomes Project for individuals of European descent. Associations of oral health symptoms reported by 21,991 individuals (mean age 63 years, 50% female, 48% never smokers), with *TAS2R* SNPs (87 in *TAS2R* genes; 37 in *TAS2R* pseudogenes; minor allele frequency > 0.01) were tested by Chi-square with Bonferroni correction and by logistic regression correcting for sociodemographic variables and oral health habits. Fifteen SNPs in *TAS2R8*, *9*, *13*, *14*, *20*, and *50* showed modest suggestive associations with self-reported sore jaw muscles. These variants were associated with relatively small changes in the odds of reporting sore jaw muscles (6 positive; 9 negative). Nine of these SNPs were in *TAS2R20* and highly correlated (r^2^ ≈ 1), likely representing a single locus-level genetic signal. However, none of the associations remained significant after Benjamini–Hochberg FDR correction across all 2604 SNP–phenotype tests, and these findings should therefore be considered exploratory. Structure-function analysis identified selected *TAS2R20* residues near the predicted ligand-binding region, providing hypotheses for future functional investigation. These exploratory findings provide hypotheses for future genetic and functional investigation and require independent replication in clinically characterized cohorts.

## 1. Introduction

Bitter Taste Receptors (T2Rs) are G-protein coupled receptors that detect bitter compounds. These receptors are encoded by genes approximately 1000 base pairs in length, referred to as *TAS2R* according to the HUGO Gene Nomenclature [1]. In humans, there are 25 functional *TAS2R* genes and 12 pseudogenes [2,3,4,5,6]. Pseudogenes are sequences that are similar to genes but cannot produce functional proteins due to genetic alterations, such as premature stop codons or frameshifts. Despite lacking protein-coding ability, growing evidence indicates that pseudogenes can still be transcribed and may play important regulatory roles in gene expression [7,8,9]. *TAS2R*s exhibit various single nucleotide polymorphisms (SNPs), which can impact receptor expression and function [10,11].

T2Rs play a crucial role in the innate immune response to oral pathogens. These receptors are expressed in various tissues, including the mucosal epithelium and immune cells [12,13,14]. T2Rs activate pathways that lead to the release of nitric oxide and calcium-mediated antimicrobial peptides, which have direct antimicrobial effects [15,16,17]. Studies have shown that genetic variants in *TAS2R*s can influence the composition of oral microbial communities [18,19]. Variations in *TAS2R*s may alter receptor expression or function, impacting the immune response to oral microorganisms and, consequently, susceptibility to oral diseases.

T2Rs can influence oral health through various mechanisms including food preferences and dietary habits, which are closely related to oral health issues such as dental caries and periodontal disease [20,21]. A previous transcriptome-wide association study identified a relationship between the expression of certain T2Rs and the occurrence of early childhood caries [22]. Furthermore, variants in *TAS2R*s are linked to alcohol consumption and smoking, which are known risk factors for periodontal diseases [23,24,25]. A previous study demonstrated that individuals with specific genotypes of *TAS2R*38 have a lower risk of periodontitis [26].

Few studies are available on the relationship between *TAS2R* genetic variants and oral health conditions. Available studies focused primarily on dental caries [18,22,26,27,28,29]. Most of these studies have concentrated on the *TAS2R*38 variants [26,27,28,29]. Research on the association between dental caries and other *TAS2R* types is limited and has yielded conflicting results [18,22]. Furthermore, the association between other oral health problems and *TAS2R* variants remains poorly understood.

The Canadian Longitudinal Study on Aging (CLSA) is a cohort study of 50,000 individuals aged 45–85 years, of whom 26,622 underwent genome-wide genotyping of DNA samples collected from blood [30,31]. Extensive demographic and clinical data on various aspects of participants’ lives have been collected to assess their impact on health and disease development [31].

In this study, we analyzed the allele distributions of *TAS2R* SNPs in the CLSA dataset. Additionally, we evaluated the association between *TAS2R* SNPs and various self-reported oral health symptoms within the cohort. To explore the structural context of selected *TAS2R20* variants, we conducted computational structure–function analyses focusing on their location relative to the predicted ligand-binding region.

## 2. Results

### 2.1. Allele Distributions of TAS2R SNPs in CLSA and 1KGP

A total of 1171 non-monomorphic variants occurring within *TAS2R* genes and pseudogenes were extracted from the CLSA. Following the application of quality control criteria, 124 SNPs and 22,974 individuals of European ancestry were included in the allele distribution analyses. Of these SNPs, 87 were located in *TAS2R* genes and 37 in *TAS2R* pseudogenes. Detailed annotations are provided in Table S1. Notably, *TAS2R20* had the highest number of SNPs (*n* = 15), followed by *TAS2R4* (*n* = 12), *TAS2R42* (*n* = 8), *TAS2R14* (*n* = 7), and *TAS2R15P* (*n* = 7). The distribution of functional consequences was as follows: missense (*n* = 38), 3′ untranslated region (UTR) (*n* = 20), synonymous (*n* = 19), 5′ UTR (*n* = 9), and stop-gained (*n* = 1; in *TAS2R19*).

**Figure 1:**
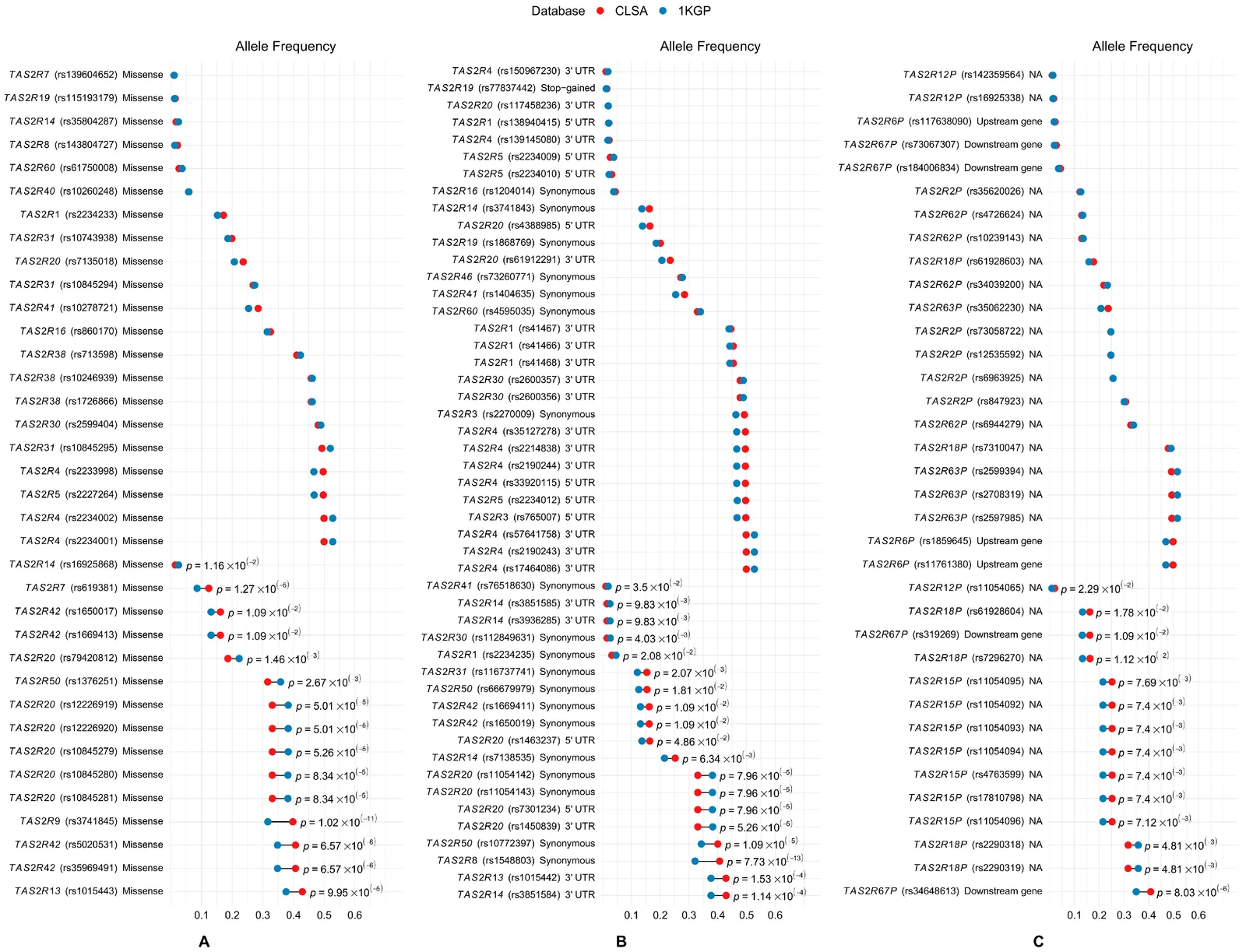
Allele distributions of *TAS2R* SNPs in CLSA and 1KGP. Dots represent the minor allele frequencies of each SNP within the European population: (**A**) Missense variants. (**B**) Non-missense variants. (**C**) Variants in *TAS2R* pseudogenes. Significant differences (adjusted *p*-value < 0.05, chi-squared test) are marked with a black line. NA: unknown functional consequence. See also Table S1.

Allele frequencies for 121 out of 124 SNPs identified in the CLSA dataset were available for the European population in 1KGP. Minor allele frequencies in CLSA and the *p*-values from the chi-squared tests comparing them with the corresponding allele frequencies in the 1KGP European population are illustrated in Figure 1 with details provided in Table S1. Chi-squared analysis revealed significant differences in allele frequency for 48 out of 121 SNPs between the two databases. Among these, 15 were missense variants, 14 were located in pseudogenes, 7 in untranslated regions, and 12 were synonymous variants. The highest proportion of variants with significantly different allele distributions was observed in *TAS2R15P*, where all seven variants exhibited differences (Table S2). *TAS2R20* also showed a substantial proportion with significant differences in 11 out of 15 variants (73%). Pairwise linkage disequilibrium between the variants is shown in heatmaps in Figure S1.

### 2.2. Prevalence of Oral Health Symptoms in the Cohort

Following quality control of the cohort with available oral health data, 21,991 individuals were included, with a mean age of 63 years. The cohort consisted of 10,920 males and 11,071 females, as summarized in Table 1. Among the participants, 8.5% identified as current smokers, 44.3% as former smokers, and 47.1% as never having smoked. Table 2 provides a summary of the prevalence of oral health symptoms in the study cohort. Only 42.6% of the population reported no oral health problems. The most frequent oral problems reported were dry mouth, affecting 17.7% of individuals, followed by questionnaire-derived periodontal disease proxy (14.6%) and decayed teeth (14.3%). Overall, 94.2% of the cohort reported their general oral health as excellent, very good, or good, while 5.8% rated it as fair or poor.

Certain conditions were significantly more prevalent in females (*p* < 0.05), including discomfort while eating due to mouth problems, swelling in the mouth, periodontal diseases, denture-related sores, dry mouth, burning mouth, painful jaw joints, and sore jaw muscles. Notably, the latter three conditions were nearly twice as prevalent in females compared to males. Conversely, some conditions were more common in males (*p* < 0.05): decayed tooth, broken tooth, toothache, bad breath, and dirty teeth or dentures. The prevalence of the remaining symptoms, including edentulism, loose tooth, and the overall presence or absence of symptoms, did not significantly differ between the sexes (*p* > 0.05).

### 2.3. Suggestive Associations Between TAS2R SNPs and Self-Reported Sore Jaw Muscles

**Figure 2:**
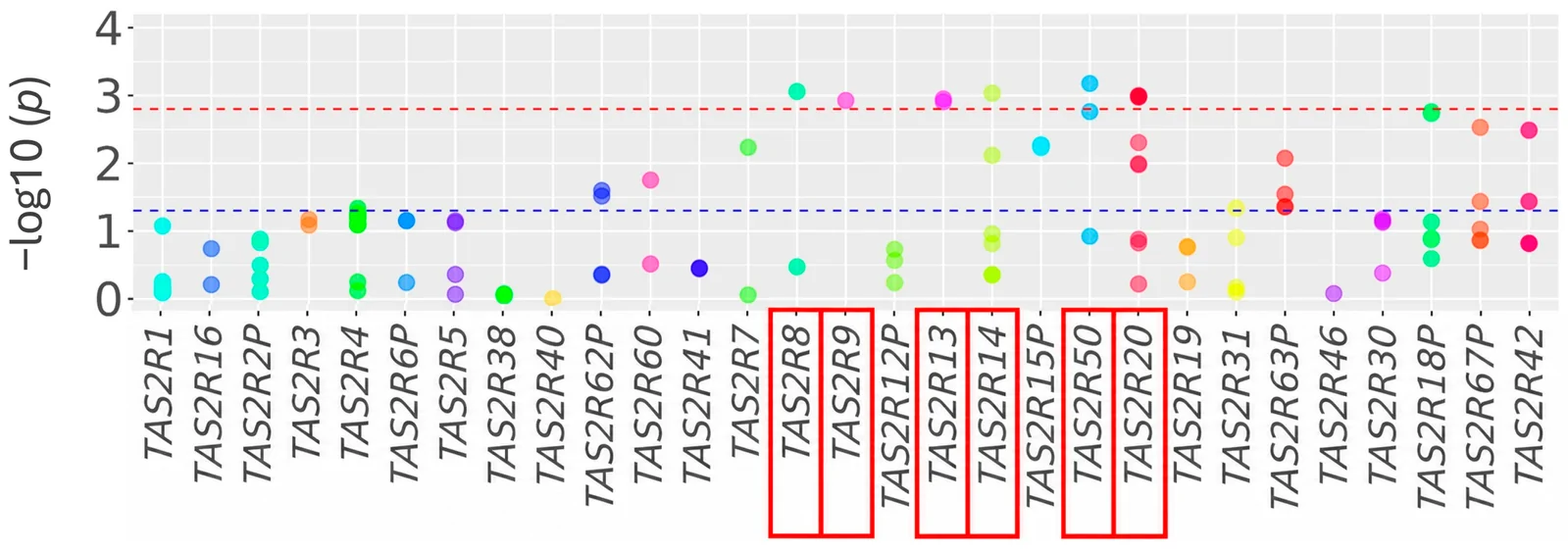
SNP-based associations between *TAS2R* variants and self-reported sore jaw muscles. Each dot represents an individual SNP, and different dot colors distinguish the *TAS2R* genes. The blue line represents the significance threshold (*p* = 0.05), while the red line marks the Bonferroni-corrected threshold, with dots above this line indicating SNPs meeting the SNP-based Bonferroni threshold (logistic regression). Genes containing these SNPs are highlighted with red rectangles. Additional Manhattan plots for other symptoms are available in Figure S2.

Based on the logistic regression analyses, controlling for sex, age, brushing frequency, smoking status, education, household income, and self-reported general health, 15 variants met the SNP-based multiple-testing threshold for association with self-reported sore jaw muscles (Table 3, Figure 2). Nine *TAS2R20* variants were in near-complete linkage disequilibrium (r^2^ ≈ 1), likely representing a single locus-level signal or haplotype, and were associated with reduced odds of sore jaw muscles. Six variants in five other *TAS2R* genes were linked to increased odds of sore jaw muscles. However, none of these associations remained significant at FDR < 0.05 when the Benjamini–Hochberg correction was applied across all SNP–phenotype tests (Table S3). No variants met the SNP-based multiple-testing threshold for association with the other oral health symptoms (Table S3). Additional plots for other symptoms are provided in Figure S2. The RNAsnp web server predicted that seven of the 15 SNPs may affect mRNA secondary structure (Figure S3).

As an exploratory cross-phenotype comparison, the genetic variants meeting the SNP-based threshold were evaluated using genome-wide association study (GWAS) summary statistics from the UK Biobank Imputed v3 dataset generated by the Neale Lab [32]. Specifically, we examined their associations with rheumatoid arthritis, an autoimmune condition that can present with sore jaw muscles if the temporomandibular joints are involved [33,34]. Six alleles spanning five genes were significantly more frequent among individuals with rheumatoid arthritis (Table S4) [32]. The remaining nine variants were all located within *TAS2R20* and were in linkage disequilibrium with one another (Table S4).

### 2.4. Structure-Function Analysis of T2R20

**Figure 3:**
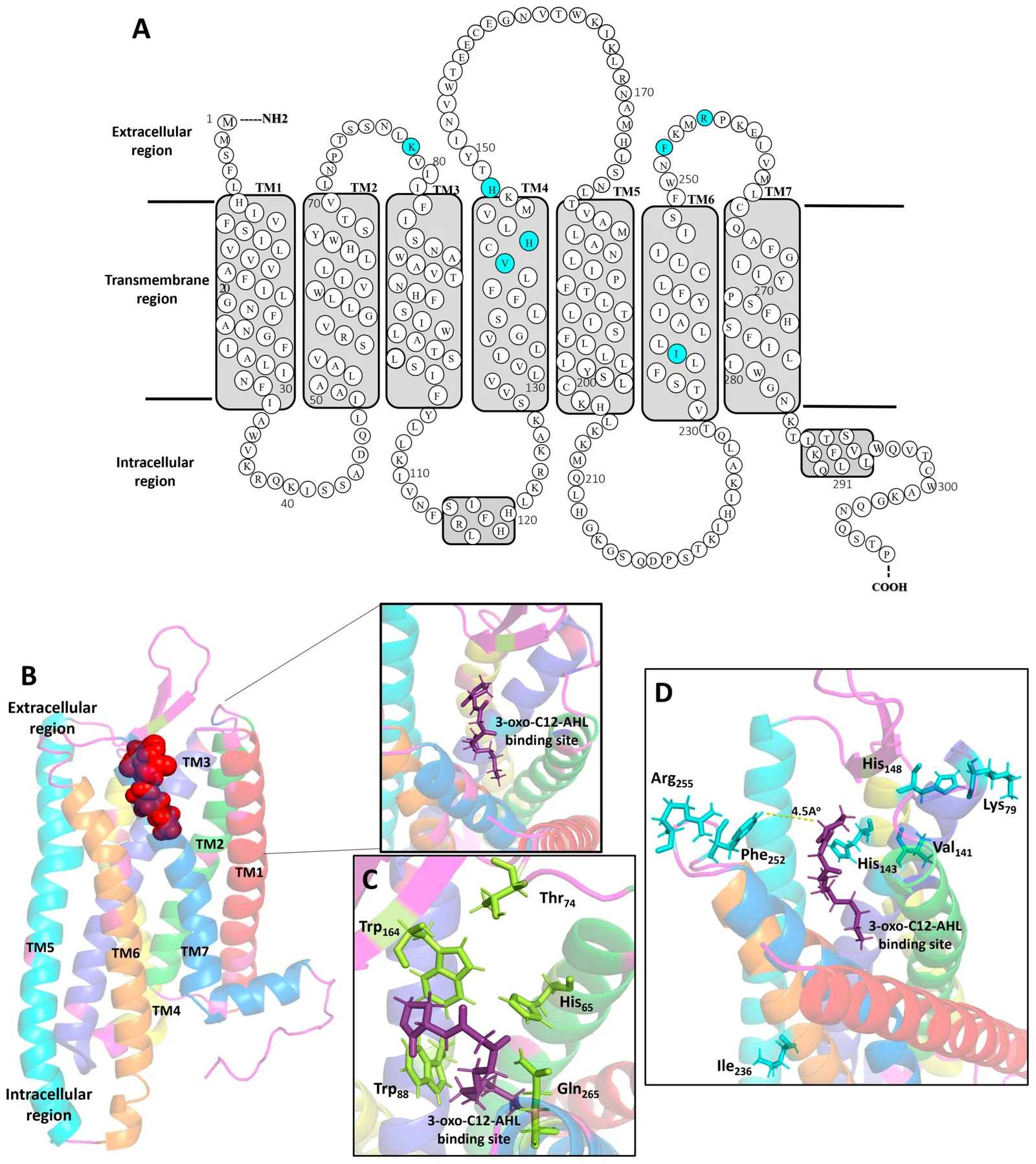
**Structure-function analysis of T2R20.** Models of T2R20 are adapted from previous studies [15,17]. (**A**) 2D model of T2R20, highlighting amino acids affected by variants in the CLSA. Underlined variants are in linkage disequilibrium. (**B**,**C**) 3D model of T2R20 from a prior publication, showing the binding site for bacterial AHL (red sphere-shaped structure), and the interacting amino acid residues [15]. (**D**) T2R20 variants in the CLSA and their proximity to the binding site. Phenylalanine 252 is the closest residue to the binding site (4.5 Å), with other variants within approximately 10 Å.

Five of the seven missense SNPs meeting the SNP-based threshold for association with sore jaw muscles were in *TAS2R20*, prompting a focus on T2R20 for structure-function analysis. Figure 3 presents 2D and 3D models of T2R20, highlighting the amino acids affected by the missense variants. The 3D model and ligand docking analysis revealed the locations of the CLSA variants within T2R20. Phenylalanine 252 was located in close proximity to the predicted binding pocket (4.5 Å), while the other variants were located approximately 10 Å from the binding site. As an exploratory structural comparison, superimposition of wild-type T2R20 models docked with levofloxacin and 3-oxo-C12-AHL showed a similar overall receptor conformation (RMSD = 0.880 Å; Figure S4). Mutant-specific modelling or docking of Phe252Ser was not performed.

## 3. Discussion

This study investigated the allele distributions of 124 common *TAS2R* variants in 25 *TAS2R* genes and 12 *TAS2R* pseudogenes within the CLSA European ancestry cohort, identifying 48 SNPs with allele distributions differing from those in the 1KGP. Fifteen variants met the SNP-based multiple-testing threshold for association with self-reported sore jaw muscles, particularly variants in *TAS2R20*; however, none remained significant after FDR correction across all 2604 SNP–phenotype tests. These associations should therefore be considered suggestive and hypothesis-generating. Structural analysis identified selected *TAS2R20* residues near the predicted ligand-binding region, providing structural context for future functional investigation.

We observed extensive variation within *TAS2R* genes, with *TAS2R20* exhibiting the highest number of variants and *TAS2R15P* showing the greatest count of variants among the pseudogenes. Our results align with those of Wooding et al., who analyzed 1KGP data from approximately 2500 individuals worldwide, excluding *TAS2R* pseudogenes [35]. They reported the highest nucleotide diversity in *TAS2R20* and the lowest in *TAS2R39*. Additionally, our results corroborate a previous study on 911 Canadian young adults, which focused on rs713598 in *TAS2R38*—a tag SNP used to identify the supertaster genotype [36]. They reported a frequency of the 49A allele at 55% among Caucasians, closely aligning with our finding of 59% among individuals of European ancestry.

The comparison of *TAS2R* genetic variants between the CLSA and 1KGP revealed generally similar allele distributions, with some notable differences, including in 11 missense variants. Six of these missense variants were in *TAS2R20*, all showing lower minor allele frequencies compared to the 1KGP. Previous studies have suggested a decrease in evolutionary pressure on human *TAS2Rs* during recent stages of evolution [35,37,38]. Therefore, these differences between the cohorts could be attributed to factors such as population structure, founder effects, genetic bottlenecks, or genetic drift [37,38,39]. Interestingly, five of the six *TAS2R20* alleles with lower frequencies in the CLSA were among the variants meeting the SNP-based threshold for association with reduced odds of self-reported sore jaw muscles. Given that these associations did not remain significant after global FDR correction, this observation should be considered exploratory and merits further investigation.

The findings of this study align with previous reports on oral health in the CLSA population [40,41]. This study identified dry mouth as the most prevalent oral health issue among older Canadian adults, with a prevalence of 15% in men and 20% in women. The questionnaire-derived periodontal disease proxy, based on self-reported gingival bleeding and/or loose teeth, was present in 14% of men and 15% of women. Since early-stage periodontal disease is often asymptomatic, self-reported symptoms may preferentially identify individuals with more apparent disease manifestations [42]. The approximately 15% prevalence observed in this study is comparable to a 2014 meta-analysis, which reported a 15–20% prevalence of severe periodontitis among North American adults aged 45 to 85 [43]. However, because the self-reported symptoms are not specific to periodontitis and no periodontal probing, clinical attachment measurements, or radiographic assessment were available, this proxy cannot be interpreted as clinically diagnosed periodontitis or used to determine disease severity.

Sex differences in oral health symptoms were notable, with females experiencing a higher prevalence of xerostomia, burning mouth syndrome, denture-related sores, sore jaw muscles, and painful jaw joints. These differences may be attributed to underlying conditions such as medication use, hormonal changes, and autoimmune disorders such as Sjögren’s syndrome, which is more common in females [44,45,46,47,48]. Decreased estrogen levels in perimenopausal women can lead to oral mucosal degeneration, increasing the risk of burning mouth syndrome [49]. Sjögren’s syndrome, an autoimmune disorder causing oral cavity dryness, predominantly affects women [46,47]. Xerostomia is also linked to *Candida* species, which can predispose individuals to denture-induced stomatitis [50]. This is consistent with the higher prevalence of denture-related sores among females. Furthermore, sore jaw muscles and painful jaw joints were twice as prevalent in females, aligning with previous studies that report a greater prevalence of temporomandibular joint disorder (TMD) among females [51,52,53]. These differences may partly be due to sex hormones, which can influence pain sensitivity [44,45]. Importantly, TMD is associated with juvenile idiopathic arthritis, an autoimmune disease that has a greater incidence in female compared to male children and can lead to deforming micrognathia and TMD in later adulthood. TMD is also seen in adult-onset rheumatoid arthritis, an autoimmune disease that is two to three times more common in women [33,34,48,54,55].

Although the questionnaire-derived phenotype used in this study does not permit a clinical diagnosis of TMD, jaw-muscle soreness is a common manifestation of TMD [56]. Based on our analyses, 15 SNPs across six genes met the SNP-based multiple-testing threshold for association with sore jaw muscles. However, none remained significant after FDR correction across all 2604 SNP–phenotype tests. These findings should therefore be considered suggestive and hypothesis-generating. Nine of these variants were located in *TAS2R20* and were in near-complete linkage disequilibrium (r^2^ ≈ 1), suggesting that they tag the same underlying genetic signal or haplotype rather than representing independent associations. As an exploratory cross-phenotype comparison, six of these alleles, spanning five genes, were also reported at different frequencies in individuals with rheumatoid arthritis in UK Biobank GWAS data [32]. The remaining variants were all located in *TAS2R20* and in linkage disequilibrium (Table S4). Among the 15 associated SNPs, we identified four synonymous and four UTR variants, consistent with growing evidence that synonymous variants are linked to various diseases and can have effects comparable to those of nonsynonymous variants [57,58]. Synonymous variants may impact mRNA structure, potentially altering its stability and translational speed, thus regulating post-transcriptional gene expression [59,60]. Indeed, four of the synonymous and UTR variants were predicted by RNAsnp to alter mRNA secondary structure; these predictions were not experimentally validated. The remaining variants were in linkage disequilibrium with missense variants (r^2^ > 0.9), suggesting that the observed SNP-based signals may reflect the same underlying genetic loci. The functional consequences of these variants remain to be experimentally determined.

Among the five missense variants in *TAS2R20* meeting the SNP-based threshold for association with reduced odds of self-reported sore jaw muscles, three were located in extracellular loops, where specific residues have been reported to interact with ligands such as antibiotics and bacterial quorum-sensing molecules [15,17]. Three-dimensional models revealed that Phe252, a residue in the third extracellular loop, is close to the predicted ligand-binding site. This spatial proximity identifies Phe252 as a candidate for future functional investigation. Mutant-specific modelling or docking of Phe252Ser was not performed; therefore, these computational observations are hypothesis-generating and require experimental validation. The Phe252Ser variant is in linkage disequilibrium with four other variants, suggesting that these variants may tag the same underlying genetic signal rather than exert independent effects. The His148Asn variant is located in the second extracellular loop at the interface with the transmembrane helix; the functional consequences of this substitution remain unknown and require experimental investigation.

TMD encompasses a range of neuromuscular and musculoskeletal disorders impacting the temporomandibular joint, masticatory muscles, and/or related tissues, resulting in symptoms such as pain, difficulty chewing, and, in severe cases, restricted jaw movement [56,61,62]. It is influenced by multiple risk factors, including malocclusion, oral parafunctions such as bruxism, psychological factors like depression and anxiety, and iatrogenic injuries [53]. Elevated inflammatory markers, including interleukins and TNF-α, have been detected in the blood, masseter muscle extracellular fluid, and saliva of individuals with TMD [52,63] and specific bacterial species, particularly *Staphylococcus aureus*, have been implicated in TMD [64,65]. Additionally, variants of genes involved in inflammation and nociception (IL-8, opiorphin) have been associated with painful TMD [66].

The SNPs meeting the SNP-based threshold for association with sore jaw muscles were in *TAS2R8*, *9*, *13*, *14*, *20*, and *50*. *TAS2R13*, *14*, *20*, and *50* are expressed at moderate to high levels in extraoral tissues [12,67]. Notably, T2R20 and T2R14 detect bacterial quorum-sensing molecules and have been implicated in immune responses [17,68]. A previous study showed that T2R14 responds to molecules from *S. aureus* by inducing inflammatory markers and inhibiting bacterial growth [69]. Several T2Rs have been reported to participate in host immune responses and the regulation of inflammatory mediators such as TNF-α and interleukins [15,16,17,68,69]. These previously reported functions provide a possible biological context for investigating the observed genetic associations. However, the present study does not establish that *TAS2R*s contribute causally to jaw muscle soreness or clinically diagnosed TMD. Given the questionnaire-derived phenotype, modest effect sizes, and lack of significance after FDR correction across all 2604 SNP–phenotype tests, these observations should be considered hypothesis-generating and require independent replication in cohorts with clinical phenotyping.

Our study found no significant association with other self-reported oral health symptoms. Research on this subject is limited and primarily focuses on dental caries and *TAS2R38* variants, which have yielded contradictory results [18,26,27,28,29]. A recent meta-analysis of genome-wide association studies also found no significant association between *TAS2R* SNPs and early childhood caries [22]. However, their transcriptome-wide analysis identified a significant association with the expression of three *TAS2R*s, including *TAS2R14*. In terms of periodontal disease, Khimsuksri et al. found a lower risk of periodontitis (pocket depth ≥ 5 mm) in individuals with the *TAS2R38* AVI non-taster genotype [26]. Conversely, Kaur et al. observed no significant association between *TAS2R* variants and the Periodontal Screening and Recording (PSR) index, which evaluates gingival bleeding and pocket depth, consistent with our findings [70]. These discrepant findings may reflect differences in periodontitis definitions. We defined periodontal disease as the presence of self-reported gingival bleeding and/or loose teeth, known features of periodontitis.

### Limitations of the Study

There are several limitations to the current study. Although the comparison with 1KGP was conducted due to its availability, the small sample sizes of subpopulations limited our ability to account for potential outliers. While we identified some differences between the datasets, exploring their evolutionary origins was not within the scope of this study. Additionally, our analyses focused on individuals of European ancestry, limiting the extension of the findings to other ethnic groups. Another limitation is that sore jaw muscle was assessed using a single self-reported item and no clinical examination, imaging, or assessment of symptom severity or duration was available. Therefore, the findings should be interpreted as associations with self-reported jaw muscle soreness rather than clinically diagnosed TMD. The self-reported data may introduce potential bias, as symptoms could not be validated through clinical exams due to the large sample size. Nevertheless, these oral health indicators have demonstrated adequate validity and reliability, and the large sample size enhances the study’s statistical power [71,72]. Another limitation is the cross-sectional design, which prevents determining causality between the variants and the symptom. This limitation could be addressed in future longitudinal studies as more CLSA follow-up data become available. Furthermore, the number of SNPs detected in different genes varied, which may contribute to the differing number of associated SNPs. This study did not account for potential interactions with environmental factors or other genetic variants. Residual confounding by factors associated with jaw muscle soreness, including psychological distress, chronic pain conditions, medication use, and unmeasured parafunctional activities, cannot be excluded. Finally, the structure–function analysis was computational and hypothesis-generating; mutant-specific modelling and functional assays were not performed, and the observed spatial relationships do not establish altered ligand affinity or receptor activity.

## 4. Materials and Methods

### 4.1. Study Population and Data Preprocessing

**Figure 4:**
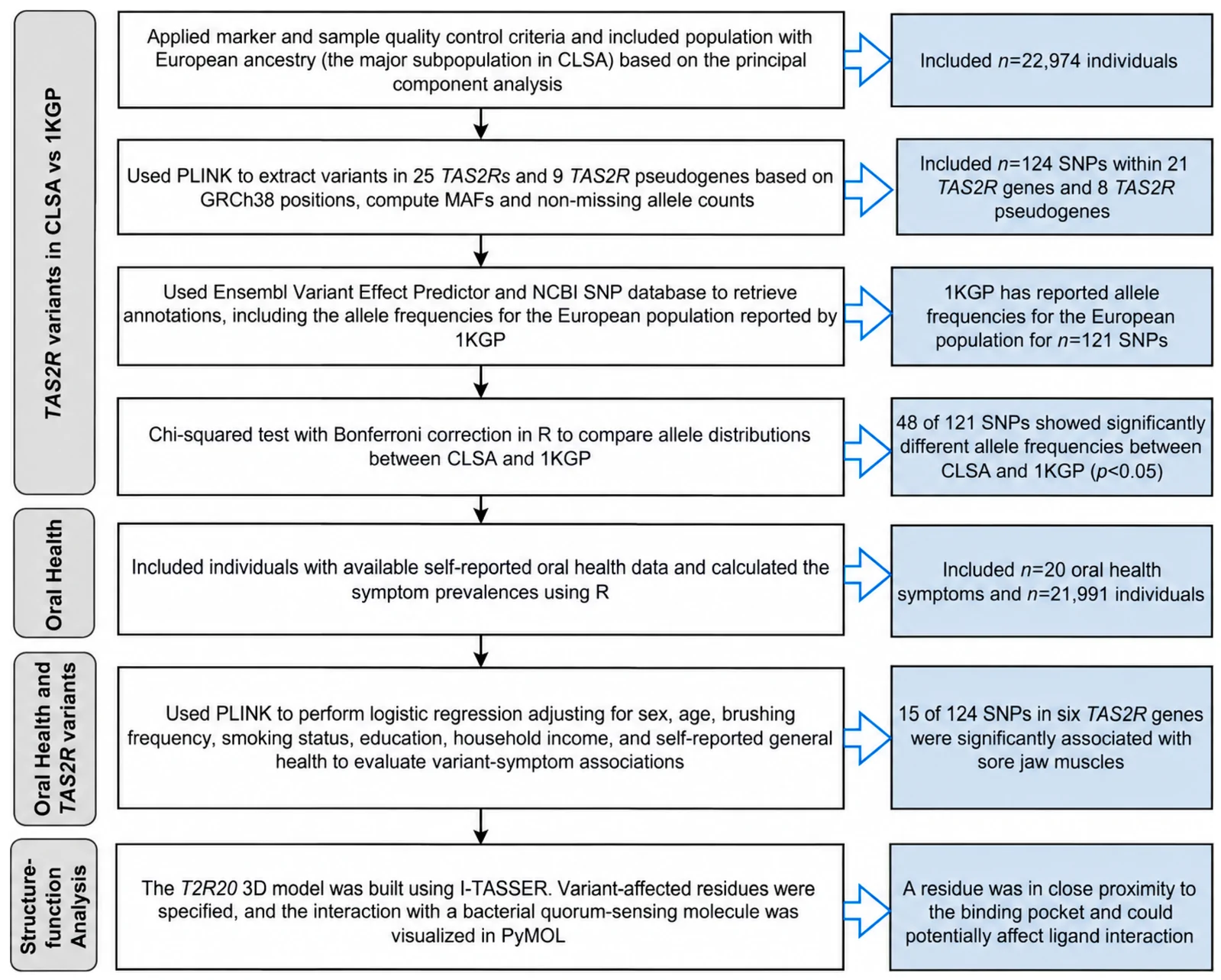
Study pipeline for analysis of *TAS2R* genetic variants in the CLSA cohort. SNP: single nucleotide polymorphism; MAF: minor allele frequency; 1KGP: 1000 Genomes Project.

The study design and statistical analysis process are summarized in Figure 4. We received annual ethics approval from the University of Manitoba’s Health Research Ethics Board (HREB HS26021-H2023:173, 20 May 2024 to 1 February 2027) and obtained access to the CLSA baseline questionnaire data (version 5) and genome-wide genetic dataset version 3 (CLSA Application ID: 2006008). Details of the CLSA protocol, including inclusion/exclusion criteria, sampling strategy, data collection, and procedures, are available for download in the Researchers section of the CLSA website (www.clsa-elcv.ca). In brief, individuals aged 45–85 at baseline were recruited from multiple sites across Canada and followed with standardized data collection, including demographics and lifestyle/behavioral measures. A subset consented to provide biosamples, including blood for genotyping. Baseline and genetic data were used for this analysis. All CLSA participants provided written informed consent to participate in the CLSA study and to the use of their data. The data used for this study is securely stored on our lab server. The genetic dataset includes information from 26,622 participants, who were genotyped for 794,409 variants using the Affymetrix Axiom array, along with an additional 308 million imputed variants from the TOPMed reference panel [30]. The positions of *TAS2R* genes in the GRCh38 genome assembly were obtained from the National Center for Biotechnology Information (NCBI) Gene database [73]. We used PLINK (v1.9) [74] to extract SNPs in *TAS2R* genes based on the GRCh38 positions. Variant annotations were obtained from the Ensembl Variant Effect Predictor (VEP) [75].

### 4.2. Eligibility Criteria

Sample and marker quality control was applied using PLINK according to protocols established in prior CLSA publications [30]. Genetic markers were excluded based on the following criteria: genotype frequency discordance between batches and across control replicates; deviation from Hardy–Weinberg equilibrium (HWE) with a *p*-value threshold of 3.15 × 10^−10^; insertions/deletions (indels); genotype missingness greater than 5%; and SNPs with minor allele frequency (MAF) ≤ 0.01. Sample exclusions were based on the following: individuals with familial relatedness of 3rd degree or closer (determined by identity-by-descent (IBD) analysis); heterozygosity outliers; discordance between self-reported and chromosomal sex; and samples with more than 5% missing genotypes. Furthermore, imputed variants with a quality score above 0.8 were included in the analysis [76]. To minimize the impact of population stratification in this study, we included only individuals of European ancestry, selected as previously described by the CLSA [30].

### 4.3. Oral Health Data in CLSA

Oral health data were obtained from the CLSA self-reported questionnaire, collected between 2011 and 2015. The CLSA oral health questionnaire, derived from the Canadian Community Health Survey 2.1, is based on self-reported oral health indicators with demonstrated good construct and concurrent validity, internal consistency, and reliability greater than 0.7 [71,77]. The questions and response values are summarized in Table S5. In addition to the 20 variables directly obtained from the questionnaire, periodontal disease was defined as the report of at least one loose tooth and/or bleeding gingiva. This definition was validated against prevalence data from the literature. For the genetic association analyses, using PLINK, categorical variables with more than two categories were dichotomized as follows: general oral health (good: excellent, very good, good vs. poor: fair, poor); and avoided eating and uncomfortable eating (yes: often, sometimes vs. no: rarely, never).

### 4.4. Statistical Analysis

#### 4.4.1. *TAS2R* Allele Distributions in CLSA and 1KGP

Allele frequencies and non-missing allele counts were calculated using PLINK for the CLSA population of European ancestry. For reference comparison, chi-squared tests were applied using R v4.3.2 (R Foundation for Statistical Computing, Vienna, Austria) [78] to compare the allelic distributions with those reported for the European population in the 1000 Genomes Project (1KGP) [79]. Bonferroni correction was applied by multiplying *p*-values by the number of independent markers in linkage equilibrium (r^2^ < 0.1 within a 1000 kb window, *n* = 35) [30]. *p* < 0.05 was considered statistically significant. Scatter plots of allele frequencies were generated using the ggplot2 package (version 4.0.3) in R [80]. Pairwise linkage disequilibrium between the SNPs was visualized using the corrplot (version 0.95) package [81].

#### 4.4.2. *TAS2R* SNPs and Oral Health Symptoms

Individuals with available oral health data were included in the genetic association analysis, with 87 excluded due to conflicting data, as they reported both an absence of natural tooth and tooth-related problems simultaneously. Individuals with missing data for any of the oral health variables were excluded from the descriptive analysis. The prevalence of oral health symptoms was summarized using R. Patients with edentulism were excluded from the analysis of tooth-related phenotypes. Logistic regression analysis was conducted using an additive genetic model in PLINK to identify variant-disease associations, adjusting for sex, age, smoking status, brushing frequency, self-reported general health, education, and household income. Individuals with missing data for the covariates were further excluded from the logistic regression analyses. Correlation analysis was performed to ensure the absence of multicollinearity between the covariates. Manhattan plots were generated using ggplot2 package to visualize the results. A total of 2604 SNP–phenotype association tests were performed (124 SNPs × 21 phenotypes). For the primary analysis, *p*-values were Bonferroni-adjusted for the effective number of independent SNPs (*n* = 35), thereby controlling the family-wise error rate across the SNP tests within each phenotype. As a sensitivity analysis addressing multiplicity across the complete SNP × phenotype testing framework, Benjamini–Hochberg FDR correction was applied to all 2604 tests. RNAsnp web server was used to predict the impact of the variants on mRNA structure [82].

### 4.5. Structure-Function Analysis

To visualize the structural locations of the amino acid residues corresponding to the variants, we used a 2-dimensional model of T2R20 from a previous publication [17], highlighting the amino acids affected by the variants. Additionally, we employed a 3-dimensional model of T2R20, constructed using I-TASSER as described in a previous study [15]. I-TASSER is a hierarchical method for predicting protein structure [83]. Energy minimization of the T2R20 structure and ligand docking with the bacterial quorum sensing molecule 3-oxo-C12-Acyl homoserine lactone (AHL) were performed using Maestro v11.0 (Schrödinger, LLC, New York, NY, USA), as previously described [15]. The interactions between T2R20 residues and AHL within the binding pocket, along with residues affected by the CLSA variants, were visualized using PyMOL molecular graphics software v3.0 (Schrödinger, LLC, New York, NY, USA). The distances of residues from the binding site were calculated using the measurement wizard in PyMOL.

### 4.6. Experimental Model and Subject Details

Participant details can be found in Table 1. The inclusion and exclusion criteria used are provided in the sections below.

## 5. Conclusions

In this study an expanded set of Bitter Taste Receptor (*TAS2R*) genetic variants in *TAS2R* genes and pseudogenes was evaluated for associations with oral health symptoms reported in Canadian adults of European ancestry. The distribution of several *TAS2R* variants differed from reference frequencies reported for European populations in the 1000 Genomes Project. Fifteen variants met the SNP-based multiple-testing threshold for association with self-reported sore jaw muscles, including nine highly correlated *TAS2R20* variants (r^2^ ≈ 1) likely representing a single locus-level genetic signal. However, none of the associations remained significant after FDR correction across all SNP–phenotype tests, and the findings should therefore be considered exploratory. Structural analyses identified selected *TAS2R20* residues near the predicted ligand-binding region, providing hypothesis-generating observations for future functional studies. Independent replication using clinically characterized phenotypes and experimental validation is required to determine the biological relevance of these findings.

## Figures and Tables

**Table 1:** Study population characteristics.

Characteristic	Males (*N* = 10,920) ^a^	Females (*N* = 11,071) ^a^	*p*-Value ^b^
Age	63 (55, 71)	62 (55, 70)	**0.001**
Smoking			**<0.001**
Never smoker	4816 (44%)	5553 (50%)	
Former smoker	5089 (47%)	4659 (42%)	
Current smoker	1013 (9.3%)	859 (7.8%)	
Missing	2	0	
Self-reported general health			**<0.001**
Excellent	2228 (20%)	2377 (21%)	
Very good	4528 (42%)	4836 (44%)	
Good	3235 (30%)	2976 (27%)	
Fair	763 (7.0%)	726 (6.6%)	
Poor	155 (1.4%)	149 (1.3%)	
Missing	11	7	
Education			**<0.001**
Less than secondary school	507 (4.7%)	610 (5.5%)	
Secondary school graduation	908 (8.3%)	1177 (11%)	
Some post-secondary education	793 (7.3%)	852 (7.7%)	
Post-secondary degree/diploma	8689 (80%)	8419 (76%)	
Missing	23	13	
Household income			**<0.001**
Less than $20,000	335 (3.2%)	638 (6.2%)	
$20,000 to less than $50,000	1761 (17%)	2657 (26%)	
$50,000 to less than $100,000	3779 (36%)	3618 (35%)	
$100,000 to less than $150,000	2356 (23%)	1826 (18%)	
$150,000 or more	2185 (21%)	1502 (15%)	
Missing	504	830	
Brushing frequency			**<0.001**
Less than once daily	1043 (9.6%)	843 (7.6%)	
Once daily	2802 (26%)	1472 (13%)	
Twice daily or more	7059 (65%)	8749 (79%)	
Missing	16	7	

^a^ Median (IQR); *n* (%); percentages are calculated using the number of non-missing observations. ^b^ Wilcoxon rank sum test; Pearson’s Chi-squared test. Significant values are bold (*p* < 0.05).

**Table 2:** Prevalence of oral health symptoms in the CLSA European ancestry cohort.

Characteristic	Males (*N* = 10,920) ^a^	Females (*N* = 11,071) ^a^	*p*-Value ^b^
General oral health			**<0.0001**
Excellent	3414 (31%)	3958 (36%)	
Very good	4407 (40%)	4318 (39%)	
Good	2399 (22%)	2210 (20%)	
Fair	545 (5.0%)	447 (4.0%)	
Poor	141 (1.3%)	120 (1.1%)	
Uncomfortable eating in last 12 months			**<0.0001**
Often	193 (1.8%)	296 (2.7%)	
Sometimes	717 (6.6%)	931 (8.4%)	
Rarely	2135 (20%)	2197 (20%)	
Never	7857 (72%)	7631 (69%)	
Avoided eating in last 12 months			**<0.0001**
Often	143 (1.3%)	213 (1.9%)	
Sometimes	399 (3.7%)	634 (5.7%)	
Rarely	1031 (9.4%)	1313 (12%)	
Never	9330 (85%)	8893 (80%)	
Edentulism	629 (5.8%)	686 (6.2%)	0.1160
Toothache	1410 (13%)	1306 (12%)	**0.0460**
Cannot chew adequately	799 (7.3%)	933 (8.4%)	**0.0013**
Swelling in mouth	465 (4.3%)	535 (4.8%)	**0.0269**
Dry mouth	1689 (15%)	2196 (20%)	**<0.0001**
Burning mouth	102 (0.9%)	207 (1.9%)	**<0.0001**
Jaw muscles sore	396 (3.6%)	788 (7.1%)	**<0.0001**
Jaw joints painful	404 (3.7%)	868 (7.8%)	**<0.0001**
Natural tooth decayed	1798 (16%)	1352 (12%)	**<0.0001**
Natural tooth loose	510 (4.7%)	522 (4.7%)	0.7087
Natural tooth broken	1278 (12%)	1124 (10%)	**0.0017**
Gums around natural teeth are sore	754 (6.9%)	894 (8.1%)	**0.0006**
Gums around natural teeth bleed	1094 (10%)	1315 (12%)	**<0.0001**
Denture-related sores	203 (1.9%)	262 (2.4%)	**0.0062**
Teeth or dentures dirty	386 (3.5%)	239 (2.2%)	**<0.0001**
Bad breath	823 (7.5%)	735 (6.6%)	**0.0258**
Periodontal disease (bleeding gums and/or loose tooth)	1508 (14%)	1700 (15%)	**0.0007**
No oral health problems	4754 (44%)	4618 (42%)	0.1600

^a^ *N* (%). ^b^ Pearson’s Chi-squared test. Significant values are bold (*p* < 0.05).

**Table 3:** *TAS2R* variants meeting the SNP-based multiple-testing threshold for association with self-reported sore jaw muscles.

rs ID	Gene	Allele ^a^	Consequence	*p* ^b^	OR [95% CI]	Predicted Effect
rs1548803	*TAS2R8*	C	Synonymous	0.031	1.16 [1.06,1.27]	LD (rs3741845 r^2^ = 0.96)
rs3741845	*TAS2R9*	A	Missense Ala187Val	0.041	1.16 [1.06,1.26]	mRNA, Protein
rs1015442	*TAS2R13*	T	3′UTR	0.039	1.16 [1.06,1.26]	mRNA, LD (rs1015443, r^2^ = 0.96)
rs1015443	*TAS2R13*	T	Missense Ser259Asn	0.043	1.15 [1.06,1.26]	mRNA, Protein
rs3851584	*TAS2R14*	G	3′UTR	0.032	1.16 [1.06,1.26]	mRNA, LD (rs1015443, r^2^ ≈ 1)
rs10772397	*TAS2R50*	C	Synonymous	0.023	1.16 [1.07,1.27]	mRNA
rs1450839	*TAS2R20*	G	3′UTR	0.036	0.85 [0.78,0.94]	mRNA, LD (r^2^ ≈ 1) ^c^
rs10845279	*TAS2R20*	A	Missense Arg255Leu	0.036	0.85 [0.78,0.94]	Protein
rs10845280	*TAS2R20*	G	Missense Phe252Ser	0.035	0.85 [0.78,0.94]	Protein
rs10845281	*TAS2R20*	C	Missense Ile236Val	0.036	0.85 [0.78,0.94]	Protein
rs12226919	*TAS2R20*	T	Missense His148Asn	0.036	0.85 [0.78,0.94]	Protein
rs12226920	*TAS2R20*	T	MissenseHis143Gln	0.036	0.85 [0.78,0.94]	mRNA, Protein
rs11054142	*TAS2R20*	A	Synonymous	0.037	0.85 [0.78,0.94]	LD (r^2^ ≈ 1) ^c^
rs11054143	*TAS2R20*	C	Synonymous	0.037	0.85 [0.78, 0.94]	LD (r^2^ ≈ 1) ^c^
rs7301234	*TAS2R20*	A	5′UTR	0.037	0.85 [0.78, 0.94]	LD (r^2^ ≈ 1) ^c^

^a^ Effect allele. ^b^ Based on logistic regression adjusting for sex, age, smoking status, tooth brushing, self-reported general health, education, and income. *p*-values were Bonferroni-adjusted for the effective number of independent SNPs (*n* = 35) within each phenotype. The odds ratio (OR) and 95% confidence intervals (95% CI) are nominal and were not adjusted for multiple comparisons. ^c^ The nine *TAS2R20* variants are in near-complete linkage disequilibrium (r^2^ ≈ 1) and likely represent a single locus-level genetic signal rather than independent associations. OR: odds ratio; CI: Confidence interval; LD: Linkage disequilibrium. Detailed LD information is provided in Figure S1, and predicted changes in mRNA secondary structures are shown in Figure S3.

## Data Availability

Resource Availability. Materials availability. This study did not generate new unique reagents. Data and code availability. • Data: This study used the CLSA genome-wide genetic data release (version 3) and Baseline Questionnaire data. Data are available from the Canadian Longitudinal Study on Aging (www.clsa-elcv.ca) for approved researchers who meet the criteria for access to de-identified CLSA data. Allele frequencies from the 1KGP were obtained through the Ensembl Variant Effect Predictor (VEP). The DOI is provided in the key resources table. • Code: The source code of this article has been deposited at https://github.com/mshafizadeh/TAS2R_OralHealth (accessed on 10 August 2026) and is publicly available as of the date of publication.

## Supplementary Materials

The following supporting information can be downloaded at: https://www.mdpi.com/article/10.3390/ijms27188128/s1.

## Abbreviations

The following abbreviations are used in this manuscript:

CLSA	Canadian Longitudinal Study on Aging
T2R	bitter taste receptor
*TAS2R*	bitter taste receptor gene
SNP	single-nucleotide polymorphism
1KGP	1000 Genomes Project
TMD	temporomandibular disorder
GWAS	genome-wide association study
AHL	N-Acyl homoserine lactone
