# Supplementary material for: Taste Genetics and Oral Health Symptoms in the Canadian Longitudinal Study on Aging
Source: Int J Mol Sci. 2026 Sep 12;27(18):8128. doi: 10.3390/ijms27188128 (PMC13606899; doi:10.3390/ijms27188128)

Table S1: Allele distributions of TAS2R SNPs in CLSA and 1KGP. Data are presented as minor allele frequencies (MAF) in CLSA European ethnicity and corresponding allele frequencies (AF) in 1KGP European population.

**rs ID**	**SNP**[a]	**Minor Allele**	**Gene**	**Consequence**	**Amino Acid Change**	**CLSA MAF**	**Non-missing Allele Count**	**1KGP AF**	**Adjusted p-value**[b]
rs41466	chr5:9627670:C:T	C	*TAS2R1*	3 prime UTR	-	0.454	45198	0.442	10.814
rs41467	chr5:9627977:A:C	A	*TAS2R1*	3 prime UTR	-	0.446	45862	0.439	20.777
rs41468	chr5:9627996:G:A	G	*TAS2R1*	3 prime UTR	-	0.455	45202	0.442	10.239
rs2234235	chr5:9629183:A:G	G	*TAS2R1*	Synonymous	Leu 284	0.032	45942	0.046	**0.021**
rs2234233	chr5:9629417:G:A	A	*TAS2R1*	Missense	Arg 206 Trp	0.171	45934	0.152	0.919
rs138940415	chr5:9630121:G:A	A	*TAS2R1*	5 prime UTR	-	0.021	45600	0.020	27.966
rs847923	chr7:12491252:G:C	C	*TAS2R2P*	NA	-	0.306	45764	0.300	20.456
rs35620026	chr7:12491542:C:T	T	*TAS2R2P*	NA	-	0.122	45798	0.125	24.972
rs6963925	chr7:12491554:C:T	T	*TAS2R2P*	NA	-	0.255	45734	0.256	34.886
rs12535592	chr7:12491615:A:G	G	*TAS2R2P*	NA	-	0.247	45814	0.247	34.600
rs73058722	chr7:12491991:A:G	G	*TAS2R2P*	NA	-	0.246	45820	0.247	35.000
rs765007	chr7:141764114:T:C	C	*TAS2R3*	5 prime UTR	-	0.498	45848	0.467	0.277
rs2270009	chr7:141764965:C:T	T	*TAS2R3*	Synonymous	Gly 269	0.493	45864	0.463	0.349
rs33920115	chr7:141777387:G:A	A	*TAS2R4*	5 prime UTR	-	0.496	45912	0.466	0.300
rs2233998	chr7:141778508:T:C	C	*TAS2R4*	Missense	Phe 7 Ser	0.497	45944	0.466	0.277
rs2234001	chr7:141778774:G:C	G	*TAS2R4*	Missense	Leu 96 Val	0.499	45910	0.528	0.472
rs2234002	chr7:141779000:G:A	G	*TAS2R4*	Missense	Asn 171 Ser	0.499	45944	0.528	0.449
rs57641758	chr7:141779930:A:G	A	*TAS2R4*	3 prime UTR	-	0.499	45894	0.528	0.427
rs139145080	chr7:141779944:A:G	G	*TAS2R4*	3 prime UTR	-	0.022	45912	0.018	9.225
rs150967230	chr7:141780098:C:T	T	*TAS2R4*	3 prime UTR	-	0.011	45794	0.018	0.184
rs35127278	chr7:141780293:G:T	T	*TAS2R4*	3 prime UTR	-	0.496	45908	0.466	0.307
rs2214838	chr7:141780413:A:G	G	*TAS2R4*	3 prime UTR	-	0.496	45906	0.466	0.307
rs2190243	chr7:141780676:C:G	C	*TAS2R4*	3 prime UTR	-	0.500	45898	0.528	0.483
rs2190244	chr7:141780772:A:G	G	*TAS2R4*	3 prime UTR	-	0.496	45908	0.466	0.307
rs17464086	chr7:141781136:G:A	G	*TAS2R4*	3 prime UTR	-	0.500	45880	0.528	0.508
rs2234009	chr7:141790231:C:T	T	*TAS2R5*	5 prime UTR	-	0.026	45948	0.038	0.058
rs2234010	chr7:141790232:G:A	A	*TAS2R5*	5 prime UTR	-	0.031	45948	0.022	0.854
rs2234012	chr7:141790307:A:G	G	*TAS2R5*	5 prime UTR	-	0.497	45878	0.468	0.406
rs2227264	chr7:141790438:G:T	T	*TAS2R5*	Missense	Ser 26 Ile	0.497	45868	0.467	0.332
rs117638090	chr7:141787930:G:A	A	*TAS2R6P*	Upstream gene	-	0.022	45912	0.018	9.060
rs1859645	chr7:141788088:A:G	G	*TAS2R6P*	Upstream gene	-	0.497	45882	0.468	0.417
rs11761380	chr7:141788474:A:C	C	*TAS2R6P*	Upstream gene	-	0.497	45882	0.468	0.417
rs619381	chr12:10801659:C:T	T	*TAS2R7*	Missense	Met 304 Ile	0.124	45924	0.086	<0.0001
rs139604652	chr12:10801984:A:T	T	*TAS2R7*	Missense	Val 196 Glu	0.011	45606	0.011	33.554
rs143804727	chr12:10806297:T:G	G	*TAS2R8*	Missense	Glu 228 Asp	0.022	45936	0.013	0.319
rs1548803	chr12:10806432:C:T	C	*TAS2R8*	Synonymous	Leu 183	0.407	45766	0.321	<0.0001
rs3741845	chr12:10809516:A:G	A	*TAS2R9*	Missense	Ala 187 Val	0.398	45938	0.316	<0.0001
rs142359564	chr12:10895096:C:T	T	*TAS2R12P*	NA	-	0.010	45908	0.013	12.386
rs16925338	chr12:10895290:T:A	A	*TAS2R12P*	NA	-	0.016	45640	0.013	10.713
rs11054065	chr12:10895780:G:A	A	*TAS2R12P*	NA	-	0.020	45946	0.009	**0.023**

rs1015442	chr12:10908096:T:C	T	*TAS2R13*	3 prime UTR	-	0.429	45930	0.377	**0.000**
rs1015443	chr12:10908523:T:C	T	*TAS2R13*	Missense	Ser 259 Asn	0.429	45938	0.376	**0.000**
rs3851584	chr12:10937478:G:T	G	*TAS2R14*	3 prime UTR	-	0.429	45904	0.377	**0.000**
rs3851585	chr12:10937794:C:G	G	*TAS2R14*	3 prime UTR	-	0.015	45942	0.025	**0.010**
rs3936285	chr12:10937839:T:A	A	*TAS2R14*	3 prime UTR	-	0.015	45942	0.025	**0.010**
rs35804287	chr12:10938607:G:A	A	*TAS2R14*	Missense	Leu 201 Phe	0.017	45942	0.025	0.468
rs3741843	chr12:10938833:C:T	C	*TAS2R14*	Synonymous	Arg 125	0.162	45912	0.136	0.074
rs16925868	chr12:10938952:T:C	C	*TAS2R14*	Missense	Thr 86 Ala	0.015	45946	0.025	**0.012**
rs7138535	chr12:10939094:T:A	A	*TAS2R14*	Synonymous	Gly 38	0.252	45916	0.215	**0.006**
rs11054092	chr12:10964560:T:C	C	*TAS2R15P*	NA	-	0.252	45838	0.215	**0.007**
rs11054093	chr12:10964615:G:C	C	*TAS2R15P*	NA	-	0.252	45838	0.215	**0.007**
rs11054094	chr12:10964693:G:A	A	*TAS2R15P*	NA	-	0.252	45838	0.215	**0.007**
rs4763599	chr12:10964722:A:G	G	*TAS2R15P*	NA	-	0.252	45838	0.215	**0.007**
rs17810798	chr12:10964833:G:C	C	*TAS2R15P*	NA	-	0.252	45838	0.215	**0.007**
rs11054095	chr12:10965032:G:C	C	*TAS2R15P*	NA	-	0.251	45830	0.215	**0.008**
rs11054096	chr12:10965197:T:C	C	*TAS2R15P*	NA	-	0.252	45798	0.215	**0.007**
rs1204014	chr7:122994789:C:T	T	*TAS2R16*	Synonymous	Thr 282	0.043	45754	0.038	8.615
rs860170	chr7:122994970:C:T	C	*TAS2R16*	Missense	His 222 Arg	0.325	45946	0.314	11.462
rs7310047	chr12:11158921:G:A	G	*TAS2R18P*	NA	-	0.478	45914	0.489	11.925
rs7296270	chr12:11158991:A:T	A	*TAS2R18P*	NA	-	0.163	45894	0.132	**0.011**
rs61928603	chr12:11159188:C:T	C	*TAS2R18P*	NA	-	0.177	45916	0.158	1.273
rs61928604	chr12:11159348:C:T	C	*TAS2R18P*	NA	-	0.162	45926	0.132	**0.018**
rs2290318	chr12:11159359:G:C	C	*TAS2R18P*	NA	-	0.316	45918	0.357	**0.005**
rs2290319	chr12:11159427:C:A	A	*TAS2R18P*	NA	-	0.316	45916	0.357	**0.005**
rs77837442	chr12:11021687:C:T	T	*TAS2R19*	Stop-gained	Trp 295 *	0.014	45918	0.012	15.228
rs1868769	chr12:11022154:G:A	G	*TAS2R19*	Synonymous	Leu 140	0.201	45770	0.186	4.040
rs115193179	chr12:11022246:T:G	G	*TAS2R19*	Missense	Lys 109 Thr	0.014	45918	0.012	15.370
rs61912291	chr12:10996791:T:G	G	*TAS2R20*	3 prime UTR	-	0.235	45812	0.206	0.100
rs117458236	chr12:10996860:C:T	T	*TAS2R20*	3 prime UTR	-	0.019	45930	0.019	35.000
rs1450839	chr12:10996933:A:G	G	*TAS2R20*	3 prime UTR	-	0.331	45948	0.383	**0.000**
rs10845279	chr12:10997112:C:A	A	*TAS2R20*	Missense	Arg 255 Leu	0.331	45948	0.383	**0.000**
rs10845280	chr12:10997121:A:G	G	*TAS2R20*	Missense	Phe 252 Ser	0.331	45946	0.382	**0.000**
rs10845281	chr12:10997170:T:C	C	*TAS2R20*	Missense	Ile 236 Val	0.331	45948	0.382	**0.000**
rs12226919	chr12:10997434:G:T	T	*TAS2R20*	Missense	His 148 Asn	0.331	45948	0.383	**0.000**
rs12226920	chr12:10997447:G:T	T	*TAS2R20*	Missense	His 143 Gln	0.331	45946	0.383	**0.000**
rs79420812	chr12:10997455:C:T	T	*TAS2R20*	Missense	Val 141 Ile	0.186	45286	0.223	**0.001**
rs11054142	chr12:10997615:G:A	A	*TAS2R20*	Synonymous	Ala 87	0.331	45942	0.382	**0.000**
rs7135018	chr12:10997641:T:C	C	*TAS2R20*	Missense	Lys 79 Glu	0.236	45904	0.207	0.098
rs11054143	chr12:10997720:T:C	C	*TAS2R20*	Synonymous	Ala 52	0.331	45942	0.382	**0.000**
rs4388985	chr12:10997952:G:A	G	*TAS2R20*	5 prime UTR	-	0.164	45900	0.138	0.090
rs1463237	chr12:10997980:C:T	C	*TAS2R20*	5 prime UTR	-	0.163	45884	0.136	**0.049**
rs7301234	chr12:10998285:G:A	A	*TAS2R20*	5 prime UTR	-	0.331	45938	0.382	**0.000**
rs2600357	chr12:11133155:G:A	G	*TAS2R30*	3 prime UTR	-	0.478	45902	0.489	12.082
rs2600356	chr12:11133203:G:T	G	*TAS2R30*	3 prime UTR	-	0.478	45902	0.489	12.082
rs2599404	chr12:11133489:A:C	A	*TAS2R30*	Missense	Leu 252 Phe	0.480	45656	0.489	15.671
rs112849631	chr12:11133780:T:C	C	*TAS2R30*	Synonymous	Thr 155	0.015	45932	0.026	**0.004**

rs116737741	chr12:11030592:T:C	C	*TAS2R31*	Synonymous	Ser 248	0.153	45870	0.120	**0.002**
rs10845294	chr12:11030687:G:C	C	*TAS2R31*	Missense	Gln 217 Glu	0.268	45796	0.273	22.105
rs10743938	chr12:11030852:A:T	A	*TAS2R31*	Missense	Met 162 Leu	0.199	45414	0.186	5.628
rs10845295	chr12:11031233:G:A	G	*TAS2R31*	Missense	Trp 35 Arg	0.493	45826	0.520	0.615
rs10246939	chr7:141972804:T:C	C	*TAS2R38*	Missense	Ile 296 Val	0.457	45922	0.461	26.426
rs1726866	chr7:141972905:G:A	G	*TAS2R38*	Missense	Val 262 Ala	0.458	45916	0.461	26.896
rs713598	chr7:141973545:C:G	G	*TAS2R38*	Missense	Ala 49 Pro	0.411	44464	0.423	10.330
rs10260248	chr7:143222638:C:A	A	*TAS2R40*	Missense	Ser 187 Tyr	0.059	45946	0.058	29.364
rs1404635	chr7:143478061:G:A	A	*TAS2R41*	Synonymous	Thr 63	0.285	45896	0.254	0.096
rs10278721	chr7:143478252:C:T	T	*TAS2R41*	Missense	Pro 127 Leu	0.285	45930	0.254	0.090
rs76518630	chr7:143478409:C:T	T	*TAS2R41*	Synonymous	Tyr 179	0.011	45746	0.019	**0.035**
rs1650017	chr12:11186007:G:C	C	*TAS2R42*	Missense	Pro 311 Ala	0.162	45944	0.131	**0.011**
rs1669411	chr12:11186008:G:A	A	*TAS2R42*	Synonymous	Asn 310	0.162	45942	0.131	**0.011**
rs1669412	chr12:11186063:T:C	T	*TAS2R42*	Missense	Arg 292 Gln	0.245	45948	NA	NA
rs1451772	chr12:11186144:C:T	C	*TAS2R42*	Missense	Tyr 265 Cys	0.245	45942	NA	NA
rs1669413	chr12:11186175:A:C	C	*TAS2R42*	Missense	Trp 255 Gly	0.162	45946	0.131	**0.011**
rs5020531	chr12:11186351:A:G	A	*TAS2R42*	Missense	Ser 196 Phe	0.406	45946	0.348	<0.0001
rs1650019	chr12:11186377:C:T	T	*TAS2R42*	Synonymous	Leu 187	0.162	45946	0.131	**0.011**
rs35969491	chr12:11186414:T:A	T	*TAS2R42*	Missense	Phe 175 Tyr	0.406	45938	0.348	<0.0001
rs73260771	chr12:11061761:C:T	T	*TAS2R46*	Synonymous	Thr 178	0.272	45920	0.277	20.458
rs10772397	chr12:10986084:C:T	C	*TAS2R50*	Synonymous	Pro 259	0.400	45946	0.343	<0.0001
rs1376251	chr12:10986253:C:T	T	*TAS2R50*	Missense	Cys 203 Tyr	0.316	45926	0.358	**0.003**
rs66679979	chr12:10986336:T:C	C	*TAS2R50*	Synonymous	Ser 175	0.154	45948	0.125	**0.018**
rs61750008	chr7:143443582:G:A	A	*TAS2R60*	Missense	Val 44 Met	0.027	45938	0.037	0.254
rs4595035	chr7:143444382:T:C	T	*TAS2R60*	Synonymous	Arg 310	0.329	45936	0.340	11.534
rs4726624	chr7:143437150:G:A	A	*TAS2R62P*	NA	-	0.129	45522	0.133	21.864
rs34039200	chr7:143437188:G:A	A	*TAS2R62P*	NA	-	0.218	45936	0.233	4.662
rs10239143	chr7:143437699:C:G	G	*TAS2R62P*	NA	-	0.129	45526	0.135	16.133
rs6944279	chr7:143437702:A:T	T	*TAS2R62P*	NA	-	0.327	45330	0.338	10.871
rs2708319	chr12:11048442:A:G	A	*TAS2R63P*	NA	-	0.493	45916	0.515	1.815
rs35062230	chr12:11048811:G:A	A	*TAS2R63P*	NA	-	0.236	45944	0.207	0.102
rs2597985	chr12:11048888:T:C	T	*TAS2R63P*	NA	-	0.493	45922	0.515	1.853
rs2599394	chr12:11049030:G:A	G	*TAS2R63P*	NA	-	0.491	45712	0.515	1.327
rs319268	chr12:11179591:T:A	T	*TAS2R67P*	Downstream gene (SMIM10L1)	-	0.245	45870	NA	NA
rs73067307	chr12:11179960:A:G	G	*TAS2R67P*	Downstream gene (SMIM10L1)	-	0.027	45916	0.019	1.431
rs319269	chr12:11179986:A:C	C	*TAS2R67P*	Downstream gene (SMIM10L1)	-	0.162	45910	0.131	**0.011**
rs34648613	chr12:11180118:T:A	T	*TAS2R67P*	Downstream gene (SMIM10L1)	-	0.407	45864	0.349	<0.0001
rs184006834	chr12:11180133:G:A	A	*TAS2R67P*	Downstream gene (SMIM10L1)	-	0.043	45736	0.036	4.081

[a] Position in GRCh38 shown as chromosome:base pair position: reference allele: alternative allele

[b] Chi-squared test with Bonferroni correction. Significant values are bolded ($p < 0.05$).

Table S2: Gene-wise count of variants with different allele distributions in CLSA compared to the 1KGP based on the adjusted chi-squared test results.

Gene	**Count of variants in CLSA**	**Count of variants with different allele distributions**[a]	**Proportion of variants with different allele distributions**[b]
TAS2R16	2	0	0.00
TAS2R19	3	0	0.00
TAS2R2P	5	0	0.00
TAS2R3	2	0	0.00
TAS2R38	3	0	0.00
TAS2R4	12	0	0.00
TAS2R40	1	0	0.00
TAS2R46	1	0	0.00
TAS2R5	4	0	0.00
TAS2R60	2	0	0.00
TAS2R62P	4	0	0.00
TAS2R63P	4	0	0.00
TAS2R6P	3	0	0.00
TAS2R7	2	0	0.00
TAS2R8	2	0	0.00
TAS2R9	1	0	0.00
TAS2R1	6	1	0.17
TAS2R67P	5	1	0.20
TAS2R30	4	1	0.25
TAS2R31	4	1	0.25
TAS2R12P	3	1	0.33
TAS2R41	3	1	0.33
TAS2R42	8	4	0.50
TAS2R50	3	2	0.67
TAS2R18P	6	4	0.67
TAS2R14	7	5	0.71
TAS2R20	15	11	0.73
TAS2R13	2	2	1.00
TAS2R15P	7	7	1.00

[a] The number of variants with significantly different allele distributions compared to the 1KGP.
[b] Count of variants with different allele distributions divided by the total count of variants in CLSA.

Figure S1: Heatmaps showing the pairwise linkage disequilibrium (LD) between *TAS2R* variants. A, B, and C represent the LD scores of variants on chromosomes 5, 7, and 12, respectively. The color intensity indicates the strength of LD, with r^2 values ranging from 0.2 to 1, while white cells represent $r^2 < 0.2$. Variants associated with sore jaw muscles are highlighted in red.

A

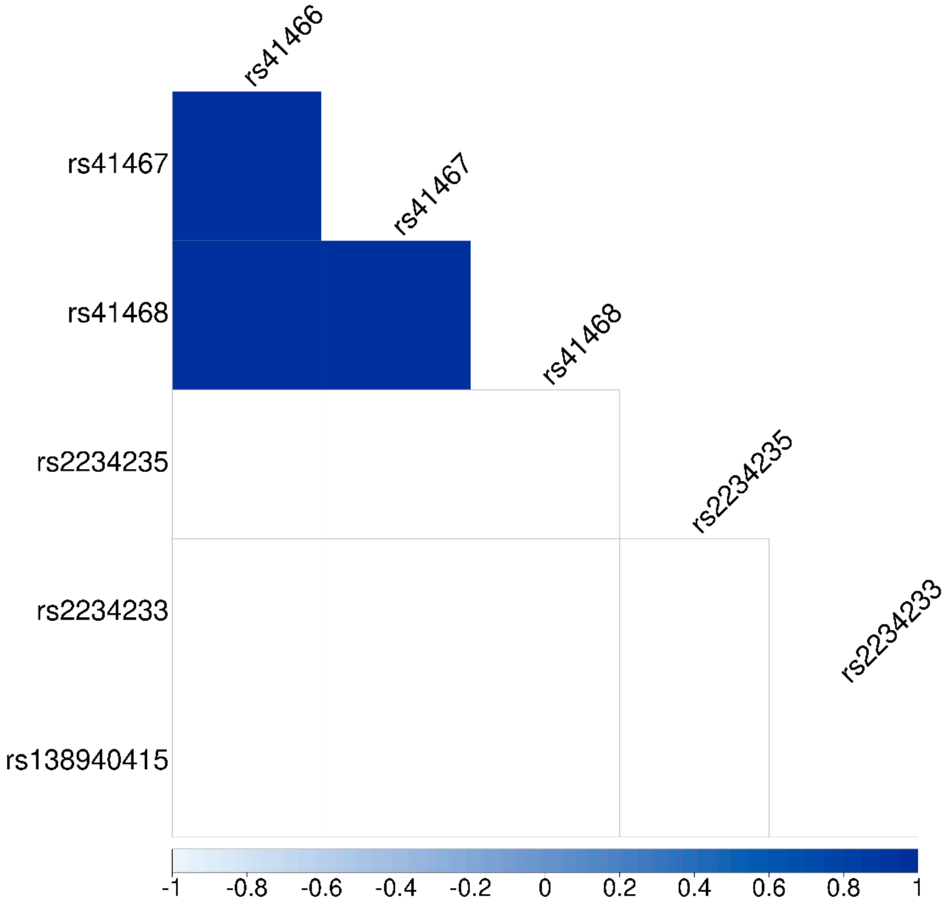

B

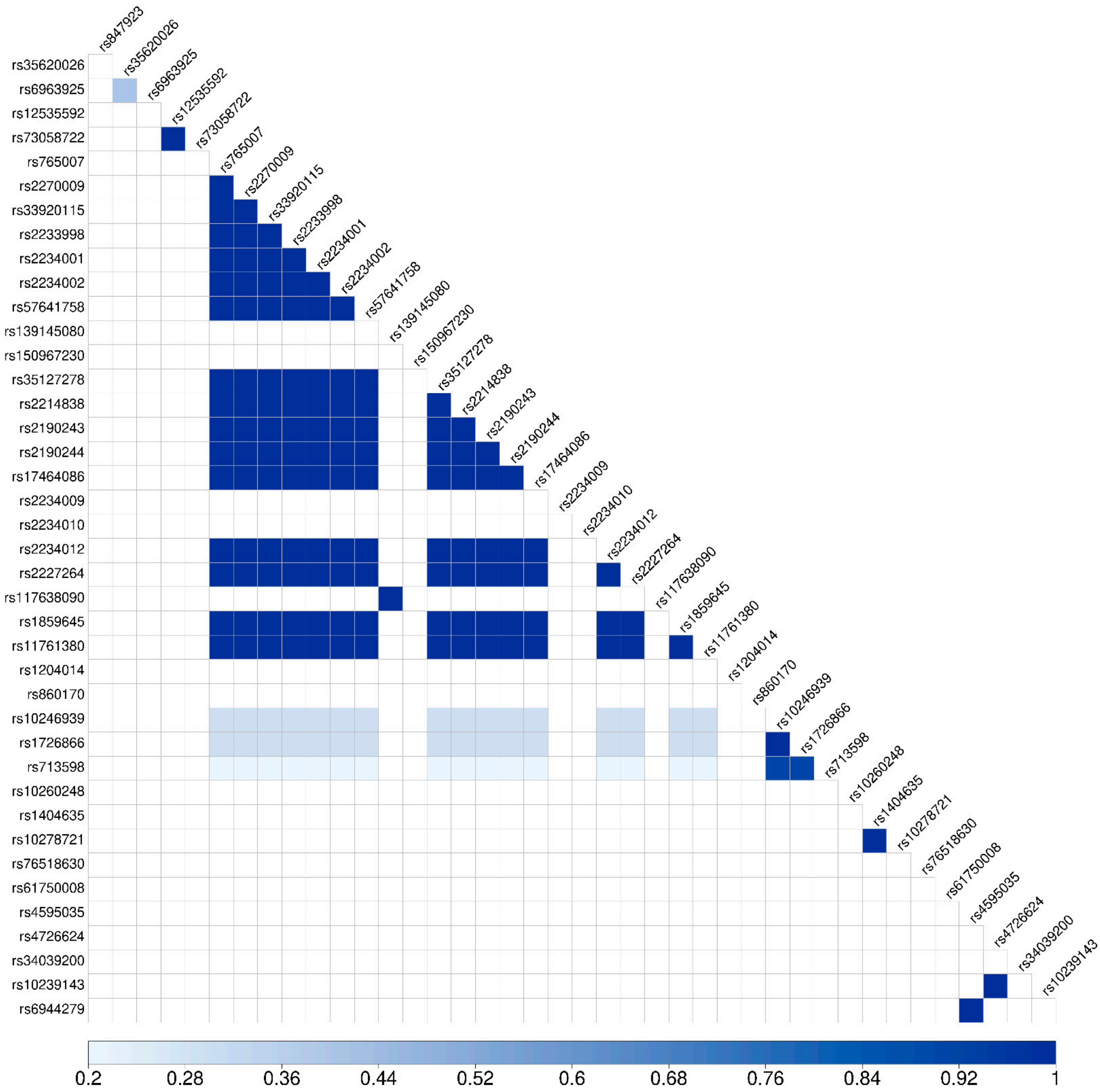
rs847923
rs35620026
rs6963925
rs12535592
rs73058722
rs765007
rs2270009
rs33920115
rs2233998
rs2234001
rs2234002
rs57641758
rs139145080
rs150967230
rs35127278
rs2214838
rs2190243
rs2190244
rs17464086
rs2234009
rs2234010
rs2234012
rs2227264
rs117638090
rs1859645
rs11761380
rs1204014
rs860170
rs10246939
rs1726866
rs713598
rs10260248
rs1404635
rs10278721
rs76518630
rs61750008
rs4595035
rs4726624
rs34039200
rs10239143
rs6944279
0.2
0.28
0.36
0.44
0.52
0.6
0.68
0.76
0.84
0.92
1

C

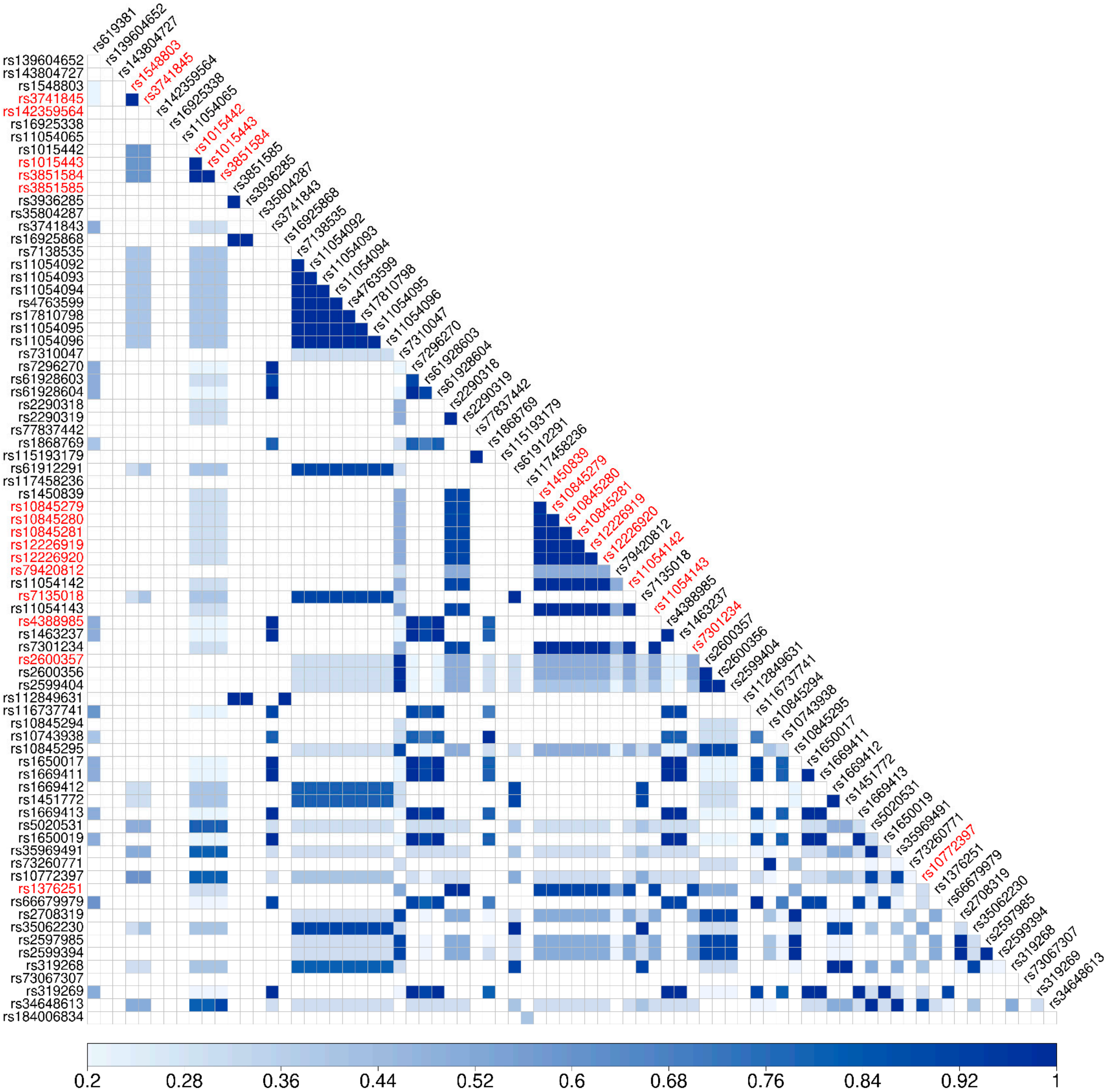
rs619381
rs139604652
rs143804727
rs1548803
rs3741845
rs142359564
rs16925338
rs11054065
rs1015442
rs1015443
rs3851584
rs3851585
rs3936285
rs35804287
rs3741843
rs16925868
rs7138535
rs11054092
rs11054093
rs11054094
rs4763599
rs17810798
rs11054095
rs11054096
rs7310047
rs7296270
rs61928603
rs61928604
rs2290318
rs2290319
rs77837442
rs1868769
rs115193179
rs61912291
rs117458236
rs1450839
rs10845279
rs10845280
rs10845281
rs12226919
rs12226920
rs79420812
rs11054142
rs7135018
rs11054143
rs4388985
rs1463237
rs7301234
rs2600357
rs2600356
rs2599404
rs112849631
rs116737741
rs10845294
rs10743938
rs10845295
rs1650017
rs1669411
rs1669412
rs1451772
rs1669413
rs5020531
rs1650019
rs35969491
rs73260771
rs10772397
rs1376251
rs66679979
rs2708319
rs35062230
rs2597985
rs2599394
rs319268
rs73067307
rs319269
rs34648613
rs184006834
0.2
0.28
0.36
0.44
0.52
0.6
0.68
0.76
0.84
0.92
1

Figure S2: Manhattan plots displaying logistic regression results. The blue line denotes the significance threshold at (p = 0.05), while the red line marks the Bonferroni-corrected threshold.

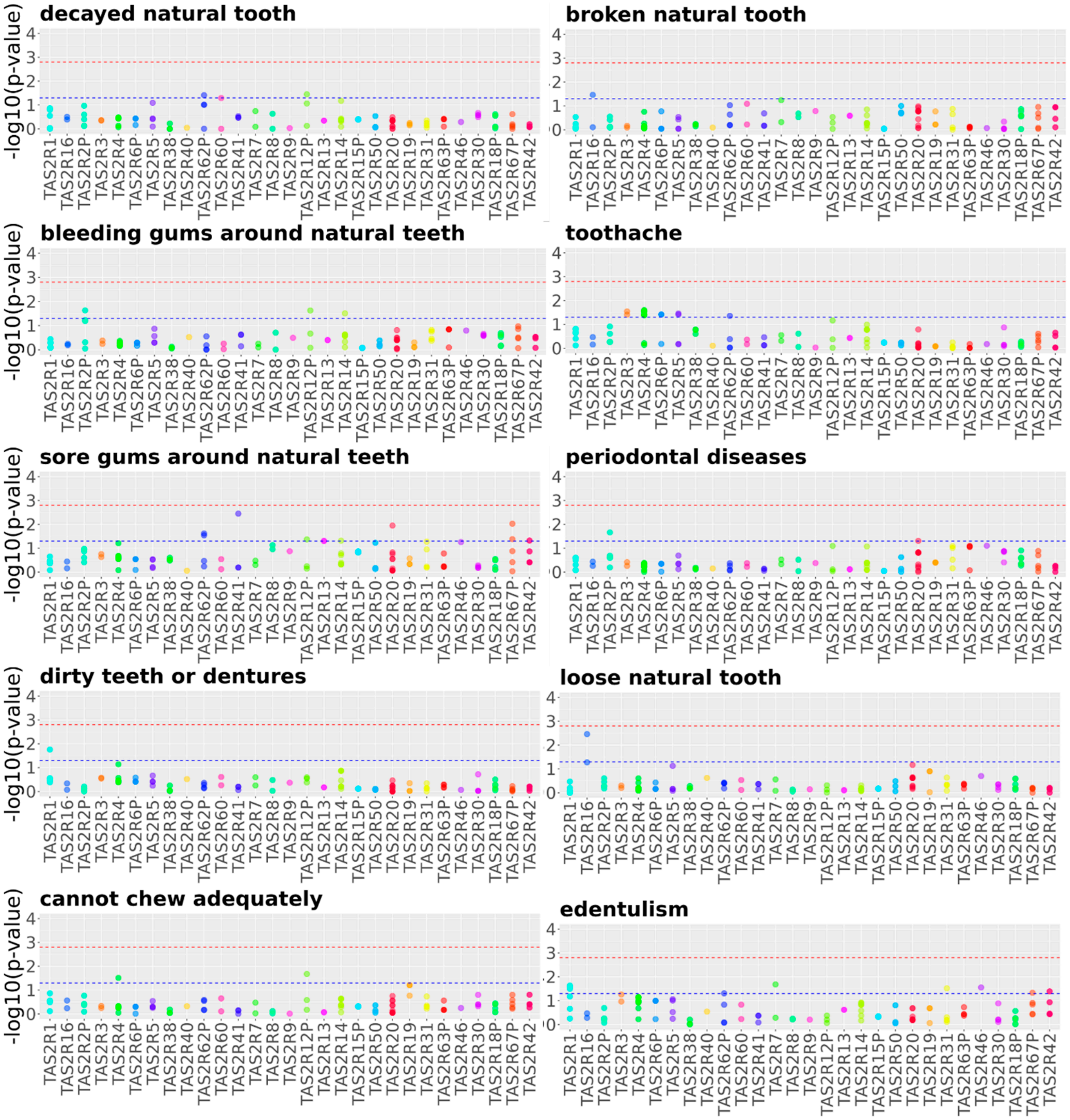

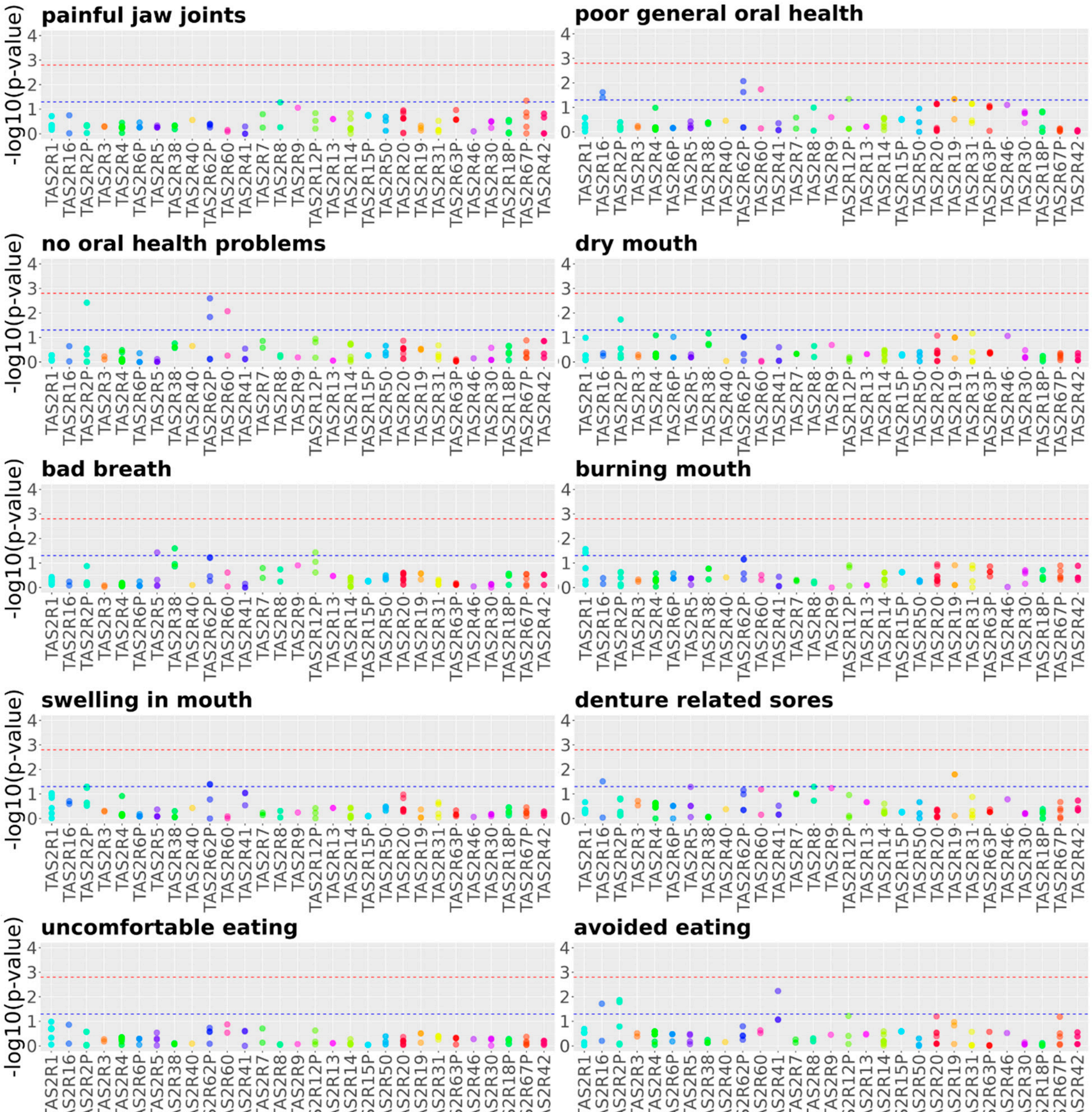

painful jaw joints
poor general oral health
no oral health problems
dry mouth
bad breath
burning mouth
swelling in mouth
denture related sores
uncomfortable eating
avoided eating
-log10(p-value)
0
1
2
3
4
TAS2R1
TAS2R16
TAS2R2P
TAS2R3
TAS2R4
TAS2R6P
TAS2R5
TAS2R38
TAS2R40
TAS2R62P
TAS2R60
TAS2R41
TAS2R7
TAS2R8
TAS2R9
TAS2R12P
TAS2R13
TAS2R14
TAS2R15P
TAS2R50
TAS2R20
TAS2R19
TAS2R31
TAS2R63P
TAS2R46
TAS2R30
TAS2R18P
TAS2R67P
TAS2R42

Figure S3: The optimal mRNA secondary structure and base pair probabilities predicted by RNAsnp. (A-B) Seven variants that may impact mRNA secondary structure. (C-D) Eight variants that are not predicted to affect mRNA structure. The upper triangle of the matrices represents base pair probabilities for the wild-type local region, and the lower triangle represents those for the mutant sequence.

A

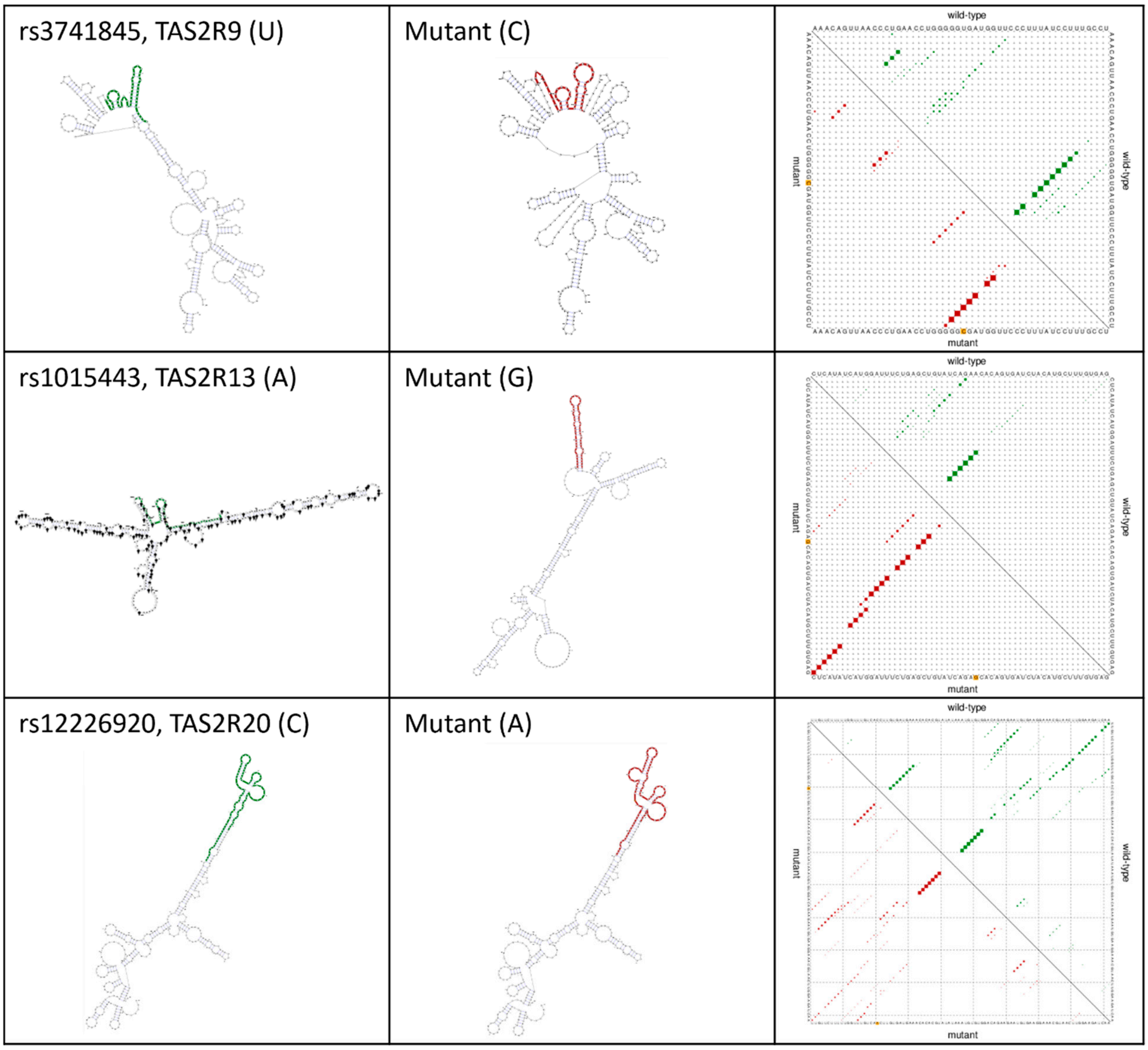

B

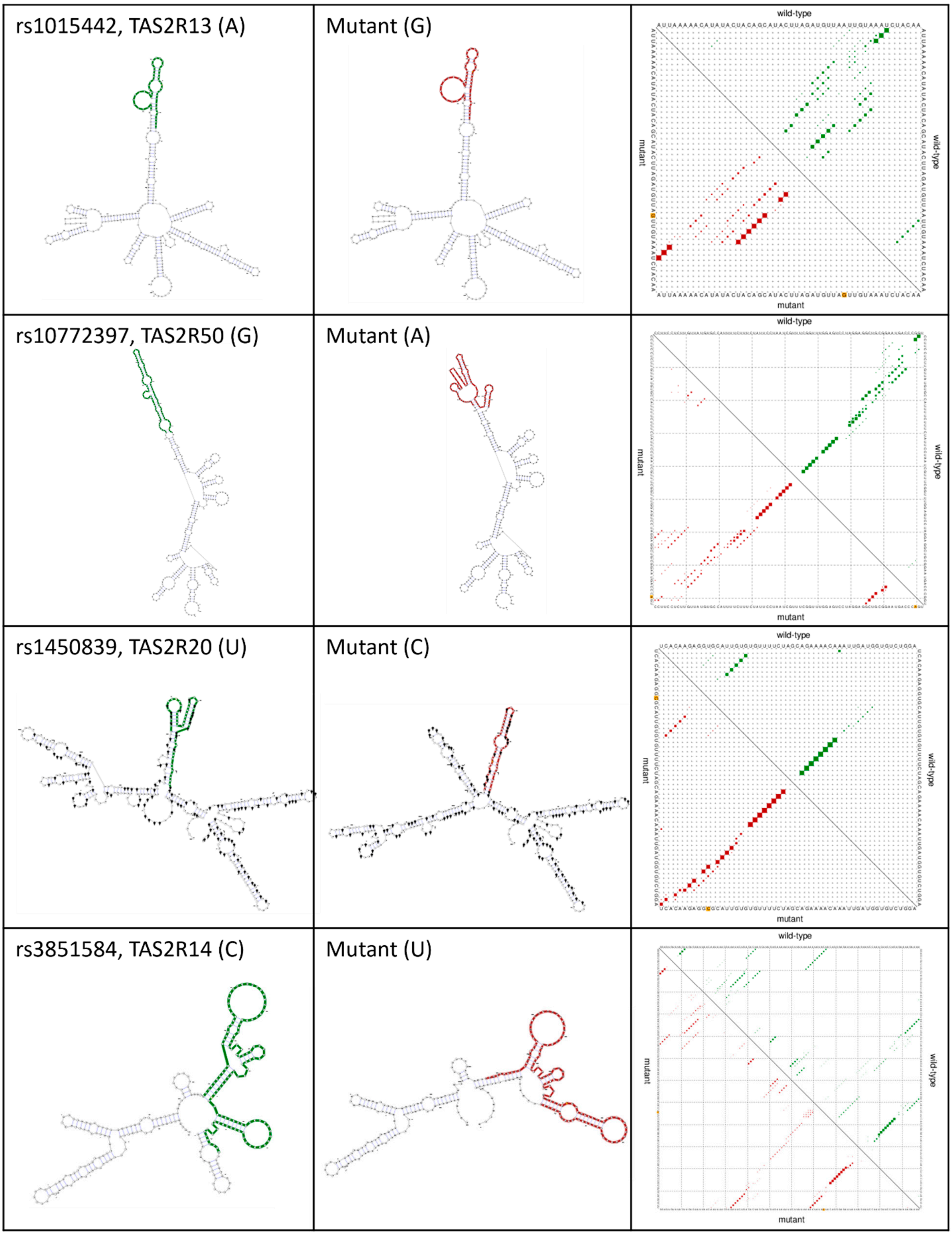
rs1015442, TAS2R13 (A)
Mutant (G)
wild-type
mutant
rs10772397, TAS2R50 (G)
Mutant (A)
wild-type
mutant
rs1450839, TAS2R20 (U)
Mutant (C)
wild-type
mutant
rs3851584, TAS2R14 (C)
Mutant (U)
wild-type
mutant

C

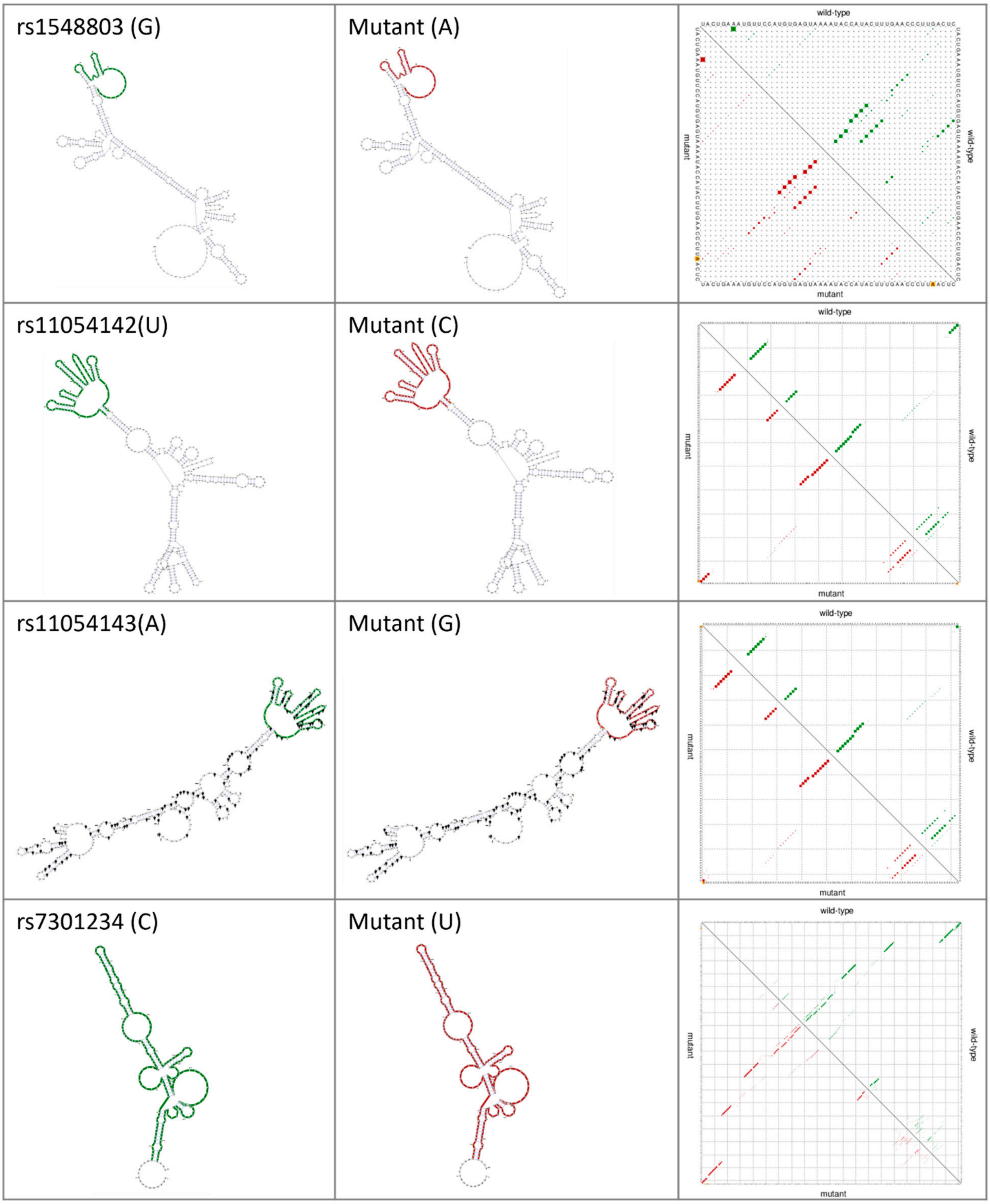
rs1548803 (G)
Mutant (A)
wild-type
mutant
rs11054142(U)
Mutant (C)
wild-type
mutant
rs11054143(A)
Mutant (G)
wild-type
mutant
rs7301234 (C)
Mutant (U)
wild-type
mutant

D

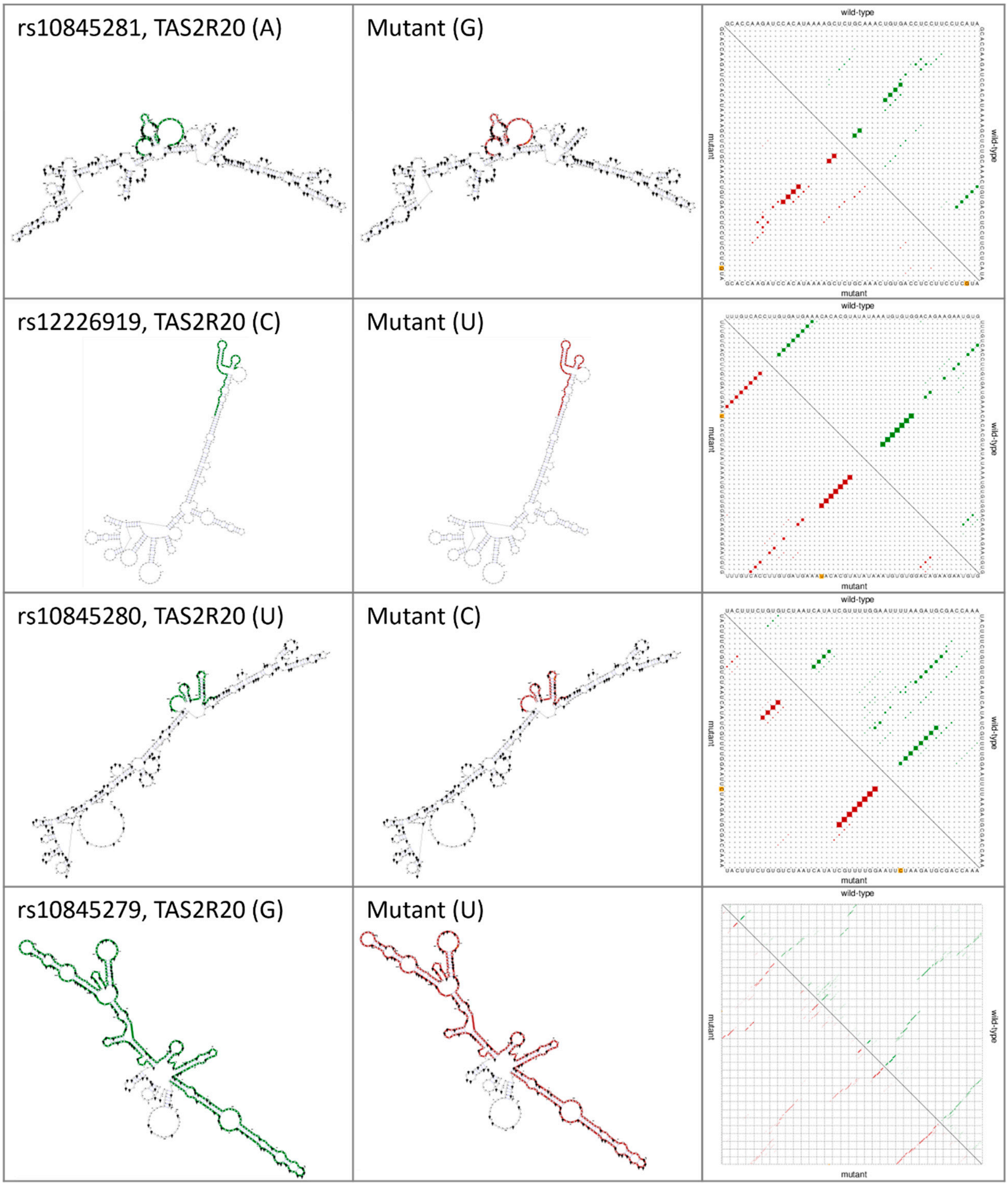
rs10845281, TAS2R20 (A)
Mutant (G)
wild-type
mutant
rs12226919, TAS2R20 (C)
Mutant (U)
wild-type
mutant
rs10845280, TAS2R20 (U)
Mutant (C)
wild-type
mutant
rs10845279, TAS2R20 (G)
Mutant (U)
wild-type
mutant

Table S4: Association of variants linked to sore jaw muscles in the current study with rheumatoid arthritis, based on genome-wide association study (GWAS) results from the UK Biobank Imputed Version 3 dataset, generated by the Neale Lab.

rs ID	Gene	MA	MAC[a]	Expected MAC[a]	Total allele count	beta	SE	P-value[b]
rs1548803	*TAS2R8*	C	4852.47	3313.85	424357	0.000774	0.000251	**0.00202**
rs3741845	*TAS2R9*	A	4933	3238.03	431173	0.000806	0.000252	**0.001361**
rs1015442	*TAS2R13*	T	4646.85	3502.98	407353	0.000665	0.000249	**0.007495**
rs1015443	*TAS2R13*	T	4649	3503.51	407305	0.000679	0.000249	**0.006297**
rs3851584	*TAS2R14*	G	4643.76	3507.59	406939	0.000674	0.000249	**0.006744**
rs10772397	*TAS2R50*	C	4874	3283.05	427125	0.000717	0.000251	**0.004253**
rs1450839	*TAS2R20*	G	2594	2556.37	229825	0.000232	0.000265	0.379467
rs10845279	*TAS2R20*	A	2596	2557.2	229900	0.000241	0.000265	0.362629
rs10845280	*TAS2R20*	G	2595	2557.28	229908	0.000233	0.000265	0.377791
rs10845281	*TAS2R20*	C	2593	2556.32	229821	0.000226	0.000265	0.392459
rs12226919	*TAS2R20*	T	2593	2554.69	229675	0.000237	0.000265	0.369795
rs12226920	*TAS2R20*	T	2594	2556.36	229825	0.000233	0.000265	0.379405
rs11054142	*TAS2R20*	A	2594	2556.36	229825	0.000233	0.000265	0.379405
rs11054143	*TAS2R20*	C	2594	2556.42	229831	0.000232	0.000265	0.380261
rs7301234	*TAS2R20*	A	2594	2556.42	229831	0.000232	0.000265	0.380261

MA: Minor allele

[a] Minor allele count in cases

[b] Statistically significant values are bolded.

Table S5: CLSA questionnaire for assessing oral health symptoms.

No.	Oral health symptoms	Answers	Question[a]
1	General oral health[b]	1=excellent, 2=very good, 3=good, 4=fair, 5=poor	In general, would you say the health of your mouth is excellent, very good, good, fair, or poor?
2	Oral health problems - Toothache	1=yes, 0=no	Have you experienced this in the past 12 months?
3	Oral health problems - Cannot chew adequately	1=yes, 0=no	Have you experienced this in the past 12 months?
4	Oral health problems - Swelling in mouth	1=yes, 0=no	Have you experienced this in the past 12 months?
5	Oral health problems - Dry mouth	1=yes, 0=no	Have you experienced this in the past 12 months?
6	Oral health problems - Burning mouth	1=yes, 0=no	Have you experienced this in the past 12 months?
7	Oral health problems - Sore jaw muscles	1=yes, 0=no	Have you experienced this in the past 12 months?
8	Oral health problems - Painful jaw joints	1=yes, 0=no	Have you experienced this in the past 12 months?
9	Oral health problems - Decayed natural tooth	1=yes, 0=no	Have you experienced this in the past 12 months?
10	Oral health problems - Loose natural tooth	1=yes, 0=no	Have you experienced this in the past 12 months?
11	Oral health problems - Broken natural tooth	1=yes, 0=no	Have you experienced this in the past 12 months?
12	Oral health problems - Sore gums around natural teeth	1=yes, 0=no	Have you experienced this in the past 12 months?
13	Oral health problems - Bleeding gums around natural teeth	1=yes, 0=no	Have you experienced this in the past 12 months?
14	Oral health problems - Denture-related sores	1=yes, 0=no	Have you experienced this in the past 12 months?
15	Oral health problems - Dirty teeth or dentures	1=yes, 0=no	Have you experienced this in the past 12 months?
16	Oral health problems - Bad breath	1=yes, 0=no	Have you experienced this in the past 12 months?
17	Oral health problems – No oral health problems	1=yes, 0=no	Have you experienced this in the past 12 months?
18	Has one or more of the original teeth	1=yes, 2=no	Do you have one or more of your original teeth?
19	Uncomfortable eating in last 12 months[b]	1=often, 2=sometimes, 3= rarely, 4= never	In the past 12 months, how often have you found it uncomfortable to eat any food because of problems with your mouth?
20	Avoided eating in last 12 months[b]	1=often, 2=sometimes, 3= rarely, 4= never	In the past 12 months, how often have you avoided eating particular foods because of problems with your mouth?

[a] Questions asked from the participants for each of the variables.

[b] For statistical analyses, categorical variables with more than two values were dichotomized as follows: general oral health: good (excellent, very good, good) vs. poor (fair, poor); avoided eating and uncomfortable eating: yes (often, sometimes) vs. no (rarely, never).

Figure S4: Structural comparison of ligand-docked wild-type T2R20 models.
Models of T2R20 are adapted from a previous study [15]. (A) Predicted wild-type T2R20–levofloxacin complex . (B) Predicted wild-type T2R20–3-oxo-C12-AHL complex. (C) Superimposition of the two ligand-docked T2R20 models, highlighting Phe249 and Phe252 and their spatial relationship to the predicted ligand-binding region. The superimposed receptor structures had an RMSD of 0.880 Å, indicating a highly similar overall receptor conformation.

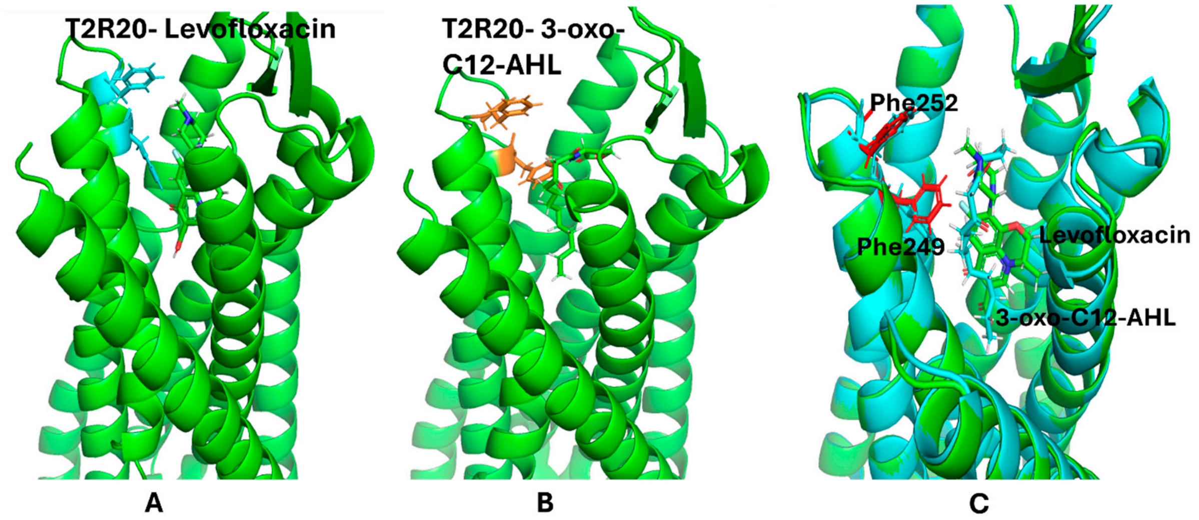

Supplement: Supplementary file 1 [file ijms-27-08128-s001.zip › ijms-4466754-supplementary.pdf]
